# An Extensive Evaluation of Read Trimming Effects on Illumina NGS Data Analysis

**DOI:** 10.1371/journal.pone.0085024

**Published:** 2013-12-23

**Authors:** Cristian Del Fabbro, Simone Scalabrin, Michele Morgante, Federico M. Giorgi

**Affiliations:** 1 Institute of Applied Genomics, Udine, Italy; 2 IGA Technology Services, Udine, Italy; 3 Center for Computational Biology and Bioinformatics, Columbia University, New York, New York, United States of America; Seoul National University College of Medicine, Korea, Republic Of

## Abstract

Next Generation Sequencing is having an extremely strong impact in biological and medical research and diagnostics, with applications ranging from gene expression quantification to genotyping and genome reconstruction. Sequencing data is often provided as raw reads which are processed prior to analysis 1 of the most used preprocessing procedures is read trimming, which aims at removing low quality portions while preserving the longest high quality part of a NGS read. In the current work, we evaluate nine different trimming algorithms in four datasets and three common NGS-based applications (RNA-Seq, SNP calling and genome assembly). Trimming is shown to increase the quality and reliability of the analysis, with concurrent gains in terms of execution time and computational resources needed.

## Introduction

Recent years have witnessed an avalanche of data produced by high throughput short read sequencing, commonly called Next Generation Sequencing (NGS). In particular, the usage of Illumina (formerly known as Solexa) reads is nowadays the basis for a series of biological applications, both in diagnostic and research fields [1,2]. These include the *de novo* assembly of genomes and transcriptomes and the alignment of short reads over an existing reference [3,4], chimeric transcript detection [5], haplotype inference [6], methylation detection [7], etc.

While *de novo* assembly is mainly used to blindly reconstruct an unknown genome or transcriptome, read alignment has several purposes: when the original material is mRNA (RNA-seq), it allows to precisely measure levels of transcripts and to identify splicing isoforms [8]. In the case of DNA-seq, read alignment is the foundation of variant detection as mismatches or gaps in the alignment reveal, if supported by an adequate number of reads, SNPs and short indels [9-12].

Illumina reads are commonly 25-250 nucleotide long sequences produced by a reversible-terminator cyclic reaction associated to base-specific colorimetric signals within the sequencing machine. Reads can be separated (“single reads”) or “paired”, in which case they are representing both extremities of the same nucleotide fragment (usually 200-1000 bp long). These colorimetric signals are translated into base calls by an internal Illumina software (CASAVA), represented in the FASTQ format [13], where each nucleotide is associated to an ASCII-encoded quality number corresponding to a PHRED score (Q) [14], which is directly translated into the probability p that the corresponding base call is incorrect by the following equation:

$$p=10^{\left(-Q/10\right)}$$

Q in recent Illumina runs ranges from 0 to 41 and therefore the error rate at each position ranges from 1 to 0.000079433. Whatever the original cause of low quality/high error chance nucleotides (e.g., air bubbles, spot-specific signal noise, malfunctioning laser/lens, etc.), the Q value is encoded and stored together with the sequence information, and this confidence information can be used for subsequent analysis, together with the sequence information itself.

Ignoring the existence of low quality base calls may in fact be detrimental for any NGS analysis, as it may add unreliable and potentially random sequences to the dataset. This may constitute a relevant problem for any downstream analysis pipeline and lead to false interpretations of data. For example, in genome assembly the inclusion of low quality reads leads to the generation of false k-mers (read substrings of fixed size) [15], which in turn increases the complexity of the subsequent assembly process [16].

There are two ways to deal with low quality nucleotides in Illumina reads. The first approach aims at correcting reads after superimposing them to each other, whereas low frequency patterns are modified based on the most frequent sequences; this approach usually works at the level of k-mers and is adopted by several preprocessing tools, such as Quake [17] and ALLPATHS-LG [18]. Some genome assembly programs can autonomously exclude lowly supported k-mers from the contig generation, e.g., ABySS [19] and ALLPATHS-LG [18]. However, the read correction approach cannot be applied wherever sequence abundance is intrinsically not uniform, such as in transcriptomics (RNA-Seq) [20], metagenomics [21] or heterogeneous cancer DNA samples [22]. Also, a low depth of coverage makes read correction strategies unfeasible, with Quake requiring at least 15x [17] and ALLPATHS-LG suggesting 100x as optimal coverage [18].

The second approach to deal with low quality nucleotides does not aim at changing the original read dataset, but rather at removing low quality bases, trying to surgically eliminate only low quality regions in a procedure known as read “trimming”. This preprocessing step is a non-trivial process, approached in different ways by different algorithms, implementations and tools, most of which are collected and described in this paper (Table 1). The basic principle of read trimming is to operate an educated estimate of read error rates trying to keep the longest possible high quality subsequence.

**Table 1:** Availability and characteristics of the trimming tools investigated in the current work.

**Tool**	**Version**	**Link**	**Language**	**Algorithm family**	**Can work directly on gzip**	**Can work on paired end**	**PHRED format autodetection**	**Works on both read ends**	**Notes**
Cutadapt	1.1	code.google.com/p/cutadapt/downloads/list	Python and C	Running sum	yes	no	no	no	Can also remove adapters, multi-threaded
ConDeTri	2.2	code.google.com/p/condetri/	Perl	Window based	yes (since v2.2)	yes	no	no	
ERNE-FILTER	1.2	sourceforge.net/projects/erne/files/	C++	Running sum	yes	yes	yes	yes	Can be combined with contaminant removal, multi-threaded
FASTX quality trimmer	0.0.13.2	hannonlab.cshl.edu/fastx_toolkit/download.html	C++	Window based	no	no	no	no	The default minimum read length parameter (-p) is set to zero
PRINSEQ	0:19:05	sourceforge.net/projects/prinseq/files/	Perl	Window based	no	no	no	yes	Also web interface for medium-size data
Trimmomatic	0.22	www.usadellab.org/cms/index.php?page=trimmomatic	Java	Window based	yes	yes	no	yes	Can also remove adapters
SolexaQA	1.13	sourceforge.net/projects/solexaqa/files/	Perl	Window based (Running sum with -bwa option)	no	no	yes	no	Cannot specify minimum read length to keep
Sickle	1.2	github.com/ucdavis-bioinformatics/sickle	C	Window based	yes	yes	no	yes	

Trimming has been broadly adopted in most recent NGS studies, specifically prior to genome assembly [23], transcriptome assembly [24], metagenome reconstruction [25], RNA-Seq [26,27], epigenetic studies [7] and comparative genomics [28]. Despite its popularity, neither a comprehensive assessment of trimming effects on common NGS analyses nor a wide comparison of the existing tools has been produced so far. Several methods have been individually described in literature [16,29,30] but their usefulness has been proven only within particular cases of genome assembly.

In the present work, we describe the existing methods for Illlumina read trimming, while introducing a novel optimized implementation of the Mott’s trimming algorithm [14]. We will assess these methods’ performances at different Q thresholds over three fundamental areas of NGS investigation: *de novo* genome assembly, RNA mapping, and genotyping (specifically, SNP identification), using two publicly available datasets for each category. We show how biological interpretation of data can be greatly influenced by the (lack of) adoption of particular trimming method/threshold combinations. Moreover, read trimming highly affects usage of resources reducing computational time and associated costs. The current work will not focus on other commonly applied Illumina read preprocessing methods, namely the already cited read correction [17], duplicate removal [31], contaminant sequence filtering [30] or adapter removal [32].

## Results

We applied the nine trimming algorithms on four different datasets (see Materials and Methods). The quality of these datasets was assessed with FastQC (see File S1 and Figure S1 for Q distribution plots) and measured by different metrics, such as the average PHRED error score, GC content biases and position-specific quality variations. The datasets vary conspicuously, possessing almost perfect quality parameters for the Yeast DNA-Seq dataset and somehow average-to-high for Lovell raw reads (Figure S1). The RNA-Seq datasets are characterized by the *Arabidopsis thaliana* reads as representative of high quality reads, while in *Homo sapiens*-derived data the error probability is both high and highly variable across read length.

### Effects of Read Trimming on Gene Expression Analysis

**Figure 1:**
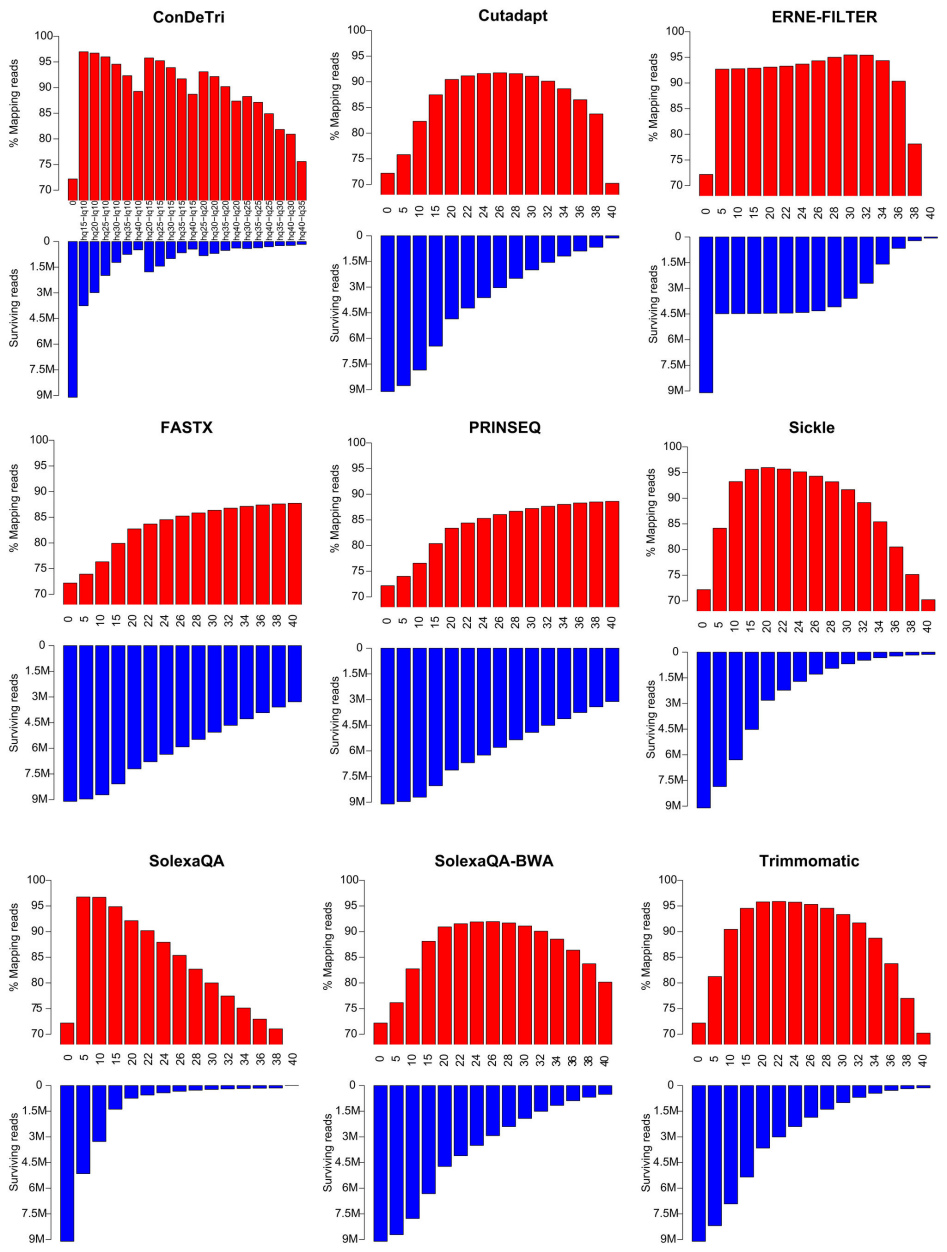
Barplots indicating the performance of nine read trimming tools at different quality thresholds on a *Homo sapiens* RNA-Seq dataset. For ConDeTri, two basic parameters are necessary, and combinations of both are reported (which explains the non-monotonic appearance of the barplots). Red bars indicate the percentage of reads aligning in the trimmed dataset. Blue bars indicate the number of reads surviving trimming.

**Figure 2:**
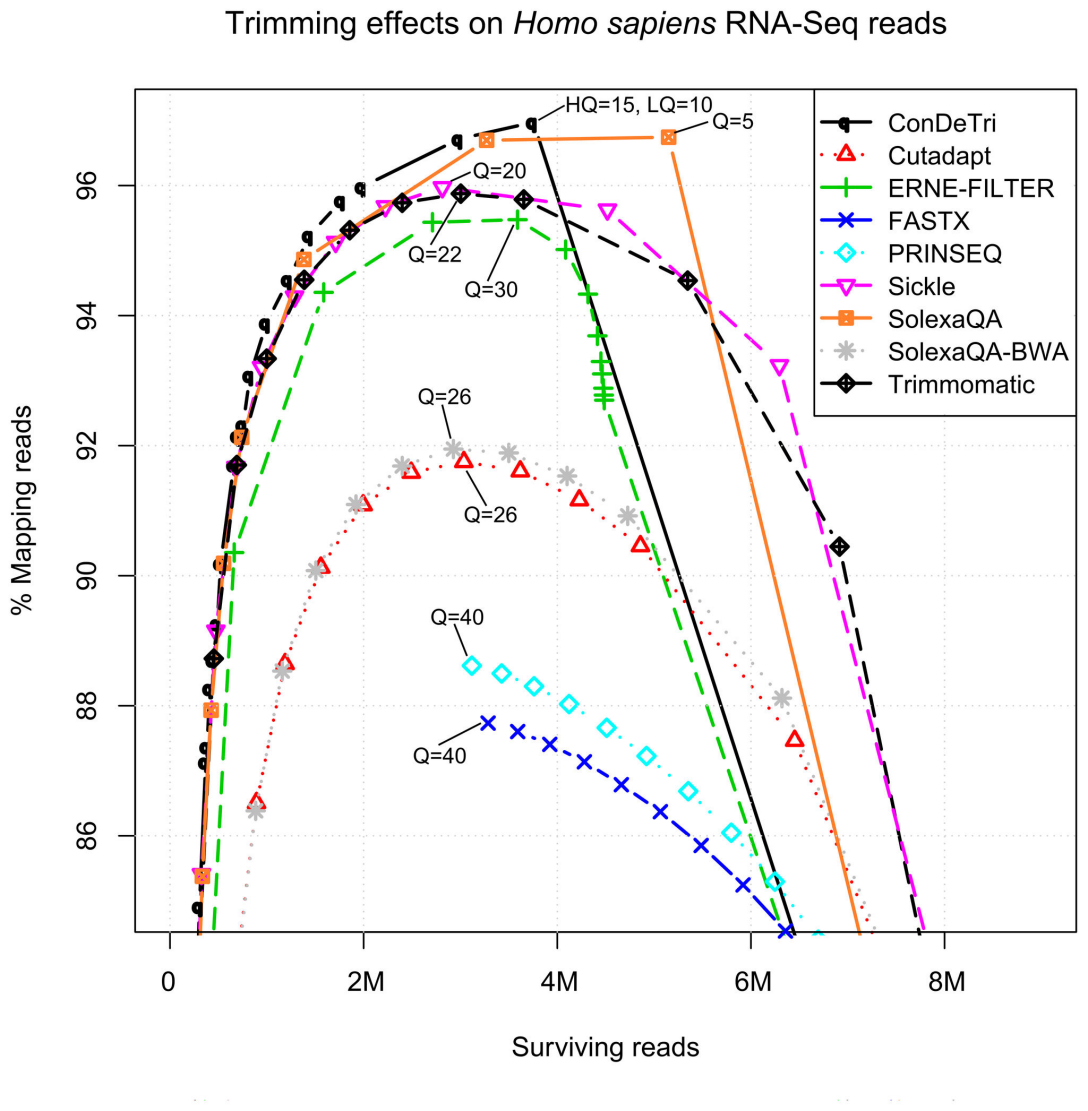
Fraction of reads mapped vs. number of reads in the quality trimmed *Homo sapiens* RNA-Seq dataset. Each symbol corresponds to a quality threshold. Peak Q parameters for each tool are reported.

We tested the performance of nine different trimming algorithms on two RNA-Seq datasets originating from human and *Arabidopsis* (see materials and methods). We assessed the number of reads and nucleotides aligning over the respective reference genomes, allowing for gap openings of the reads over spliced regions. It is evident how the trimming process in all cases reduces the number of reads, while increasing the percentage of the surviving dataset capable of correctly aligning over the reference genome. In the case of the low quality *Homo sapiens* dataset (Figure 1), while 72.2% of the untrimmed dataset reads are aligned, the trimmed ones reach values above 90%, with peaks in ConDeTri at 97.0% (HQ=15, LQ=10) and SolexaQA (Q=5) at 96.7% (Table 2). However, SolexaQA achieves the highest quality while keeping the highest number of reads, and therefore seems to be the optimal tool to maximize the tradeoff between loss of reads and increase in quality, at least in low quality RNASeq datasets such as the one analyzed here (Figure 2). For this dataset, we could observe a pseudo-optimal tradeoff between read loss and quality of the remaining reads, expressed as number of reads aligned over the total number of reads (Figure 1), which is between Q=20 and Q=30 for SolexaQA-BWA, Trimmomatic, Sickle, Cutadapt and ERNE-FILTER. Other trimmers, such as FASTX, being able to operate only from 3’end, do not achieve the same performance as the other tools (Figure 2). While retaining a similar ratio of correctly mapped reads (roughly assessed by the percentage of reads mapping within UCSC gene models), the loss of information is consistent when compared to untrimmed datasets (Figure S2). 

**Table 2:** Summary of comparisons between the trimming tools investigated in this study.

	RNASeq		Genotyping		Genome Assembly					
	Arabidopsis dataset	Human dataset	Yeast dataset	Peach dataset	Yeast dataset			Peach dataset		
	Max %Mapped Reads (threshold)	Max %Mapped Reads (threshold)	APOMAC at default threshold	APOMAC at default threshold	N50 (bp)	Accuracy	Recall	N50 (bp)	Accuracy	Recall
Untrimmed	82.774%	72.189%	0.2367%	0.2909%	9,095	99.196%	92.734%	18,093	95.116%	74.272%
ConDeTri	98.980% (HQ=40,LQ=35)	96.973% (HQ=15,LQ=10)	0.0485%	0.0851%	4,830	99.600%	91.834%	14,525	96.389%	75.090%
Cutadapt	99.422% (Q=40)	91.751% (Q=26)	0.0647%	0.1589%	6,256	99.692%	92.874%	17,653	95.349%	74.466%
ERNE-FILTER	98.687% (Q=38)	95.475% (Q=30)	0.0638%	0.1564%	6,214	99.691%	92.863%	17,665	95.374%	74.482%
FASTX	98.733% (Q=40)	87.733% (Q=40)	0.0655%	0.1614%	6,357	99.692%	92.892%	17,692	95.399%	74.510%
PRINSEQ	98.752% (Q=40)	88.616% (Q=40)	0.0652%	0.1599%	6,357	99.692%	92.890%	17,690	95.345%	74.465%
Sickle	99.422% (Q=40)	95.971% (Q=20)	0.0547%	0.1308%	5,382	99.446%	92.194%	17,074	95.495%	74.504%
SolexaQA	99.002% (Q=40)	96.743% (Q=5)	0.0644%	0.1581%	3,209	99.642%	89.770%	13,571	96.223%	74.490%
SolexaQA-BWA	98.705% (Q=38)	91.947% (Q=26)	0.0409%	0.0645%	6,256	99.692%	92.875%	17,662	95.328%	74.449%
Trimmomatic	99.422% (Q=40)	95.875% (Q=22)	0.0511%	0.1119%	4,784	99.579%	91.851%	16,141	95.766%	74.629%

By default threshold (genotyping and genome assembly columns) we set HQ=25,LQ=10 for ConDeTri, and Q=20 for the other tools. APOMAC: Average Percentage Of Minor Allele Calls (see Materials and Methods). Accuracy: percentage of genome assembly that could be mapped to the reference genome. Recall: percentage of reference genome covered by the assembly.

It is interesting to note that in general every tool shows different optimal Q thresholds (Figure 2 and Table 2) for maximizing the quality of the trimmed reads (expressed in this case by percentage of mapping reads over the reference). Also, every tool shows different trends between Q and mappability (percentage of post-trimming reads mapped on the reference genome): for some (such as SolexaQA and ConDeTri) loose thresholds are enough to achieve the most robust output. For other (such as FASTX and PRINSEQ), the highest possible threshold seems the optimal solution in terms of quality (with a concurrent loss of reads). Finally, some tools (like Cutadapt, Sickle, SolexaQA-BWA and Trimmomatic) possess an ideal intermediate Q threshold maximizing the relative amount of surviving reads alignable on the reference genome. In the case of the higher quality dataset originating from *Arabidopsis thaliana*, all tools have a comparable performance and no clear identifiable best Q for tradeoff between mappability and read loss. Starting from an untrimmed baseline of mappability of 82.8%, all tools reach a mappability of above 98.5% with stringent thresholds (Q>30, see Table 2 and Table S1). In both cases however, trimming affects and removes the most “unmappable” parts of the dataset, already at lower thresholds. Carrying a trimmed but reliable subset of the original RNA-Seq reads can reduce the need for disk space and the time needed for the overall alignment process, as high-error sequences would have already been eliminated.

### Effects of read trimming on SNP identification

**Figure 3:**
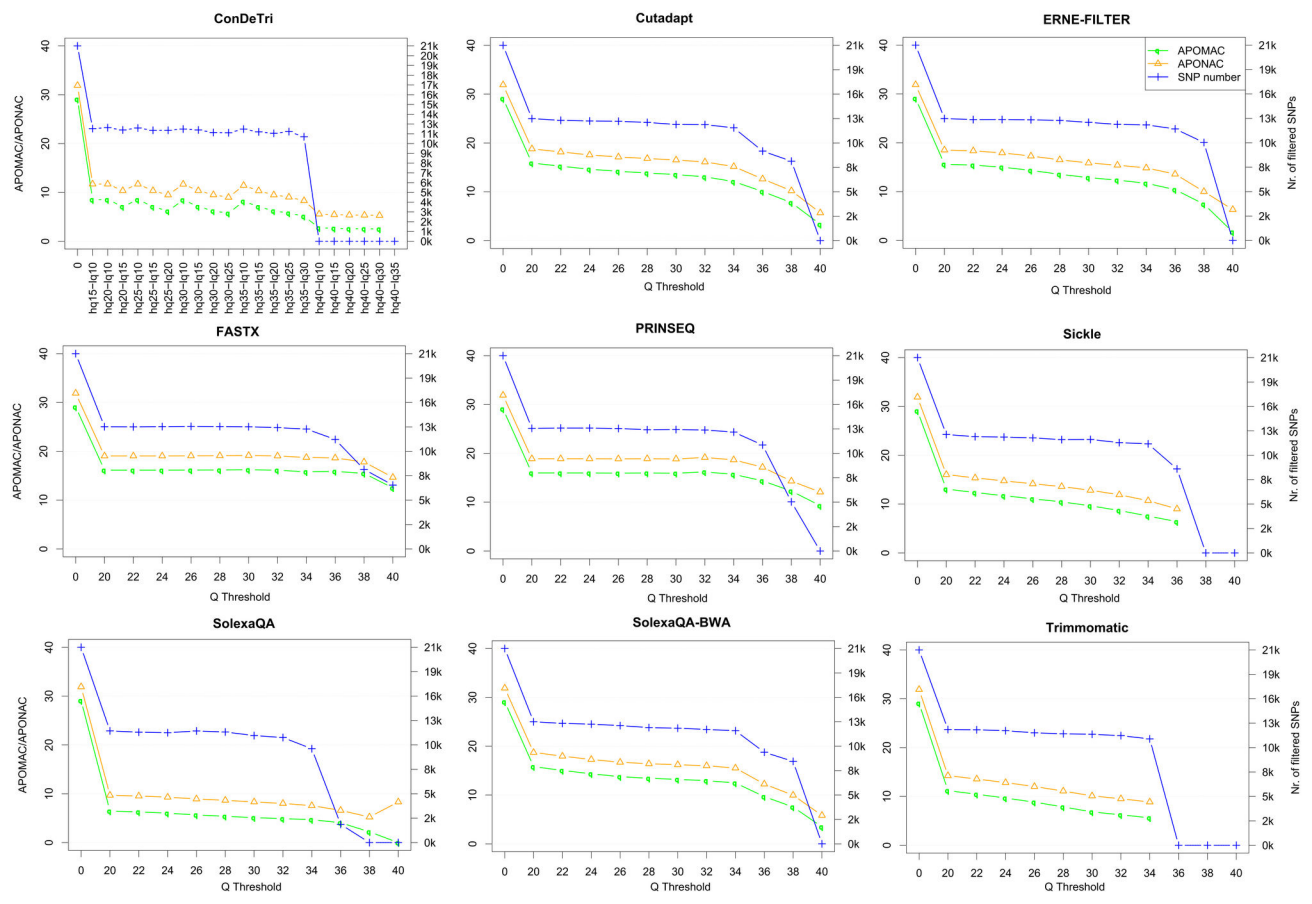
Comparative assessment of variant detection based on *Prunus persica* reads aligned on the reference peach genome. Several read trimming method/threshold combinations are tested. The Average Percentage of Minor Allele Call (APOMAC) or of Non-reference Allele Call (APONAC) are reported, together with the total number of high-confidence SNPs.

**Figure 4:**
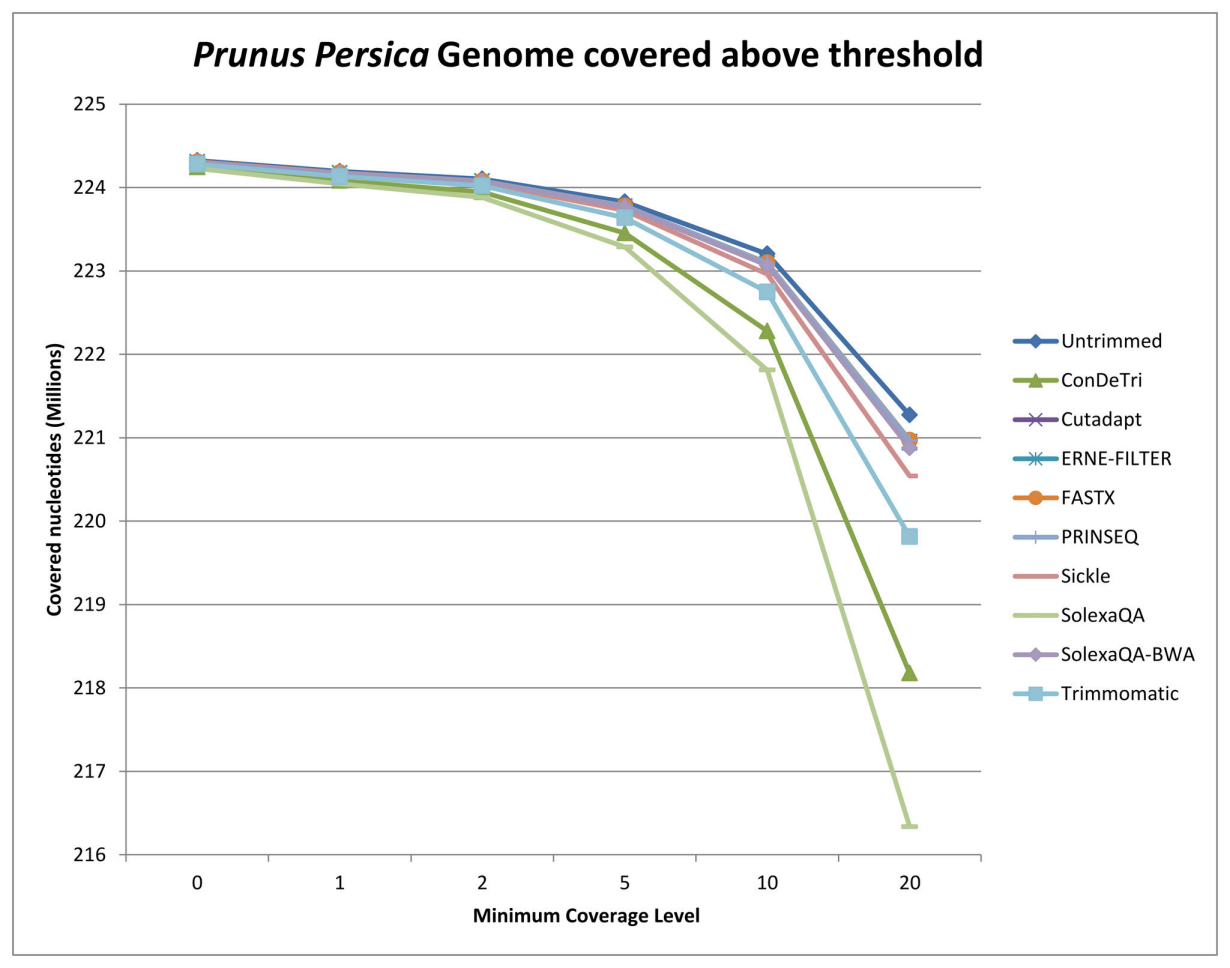
Number of covered nucleotides in the *Prunus persica* genome (total size: 227M bases) above minimum coverage thresholds. The analysis was performed on untrimmed reads and after trimming with 9 tools at Q=20 (for ConDeTri, default parameters HQ=25 and LQ=10 were used).

In order to assess the impact of trimming on SNP identification we used reads originating from dihaploid genome samples, specifically from the *Prunus persica* Lovell variety and from the *Saccharomyces cerevisiae* YDJ25 strain. In such genetic backgrounds, it is possible to evaluate any non-homozygous nucleotide call as a direct estimate of false positive SNP calling. In order to do so, we assessed the Average Percentage of Minor Allele Calls as an index termed APOMAC. At the same time, we measured the Average Percentage of Non-reference Allele Calls APONAC), although the latter is an underestimation of APOMAC, since it assumes that the sequenced individual has a genome identical to the reference sequence. The total non-homozygous nucleotide presence, related to false positive SNP calling and assessed by the APOMAC index, is -as expected- reduced by trimming (Figure 3). All trimmers drastically reduce the percentage of alternative allele nucleotides aligned over the reference genomes, both in *Prunus persica* (Figure 3) and in yeast (Table 2 and Table S1), bringing this false positive call indicator from 30% to 10% or less of the total aligned nucleotides. This rather spectacular loss of noise can be achieved with any trimmer with a Q threshold equal to or above 20 (Table S1). Best performing tools, in terms of APOMAC and APONAC, are ConDeTri and SolexaQA, which quickly reduce the number of minor allele calls. While increasing the quality of SNP calling, the coverage loss due to trimming is minor: FASTX, SolexaQA-BWA, PRINSEQ, Cutadapt and ERNE-FILTER at default Q values all process the reads without a noticeable loss of covered reference genome. This has been tested and reported by different minimum coverage thresholds (Figure 4).

### Effects of read trimming on de novo genome assembly

**Figure 5:**
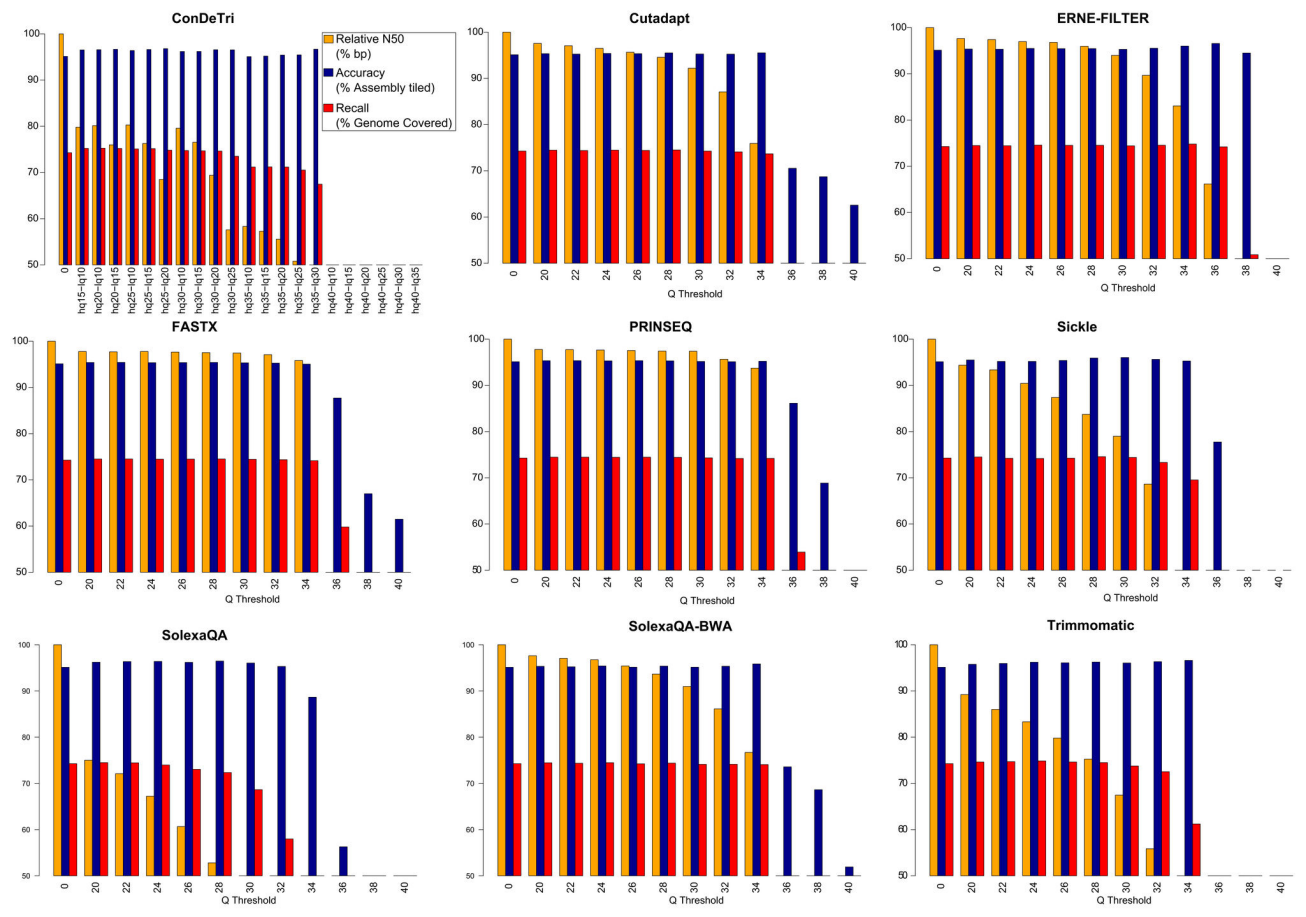
Comparative assessment of genome assembly metrics based on *Prunus persica* reads. Several read trimming method/threshold combinations are tested. Yellow bars report the N50 (relative to the untrimmed dataset N50). Blue bars report the accuracy of the assembly (% of the assembled nucleotides that could be aligned on the reference *Prunus persica* genome). Red bars report the recall of the assembly (% of the reference *Prunus persica* genome covered by the assembly).

**Figure 6:**
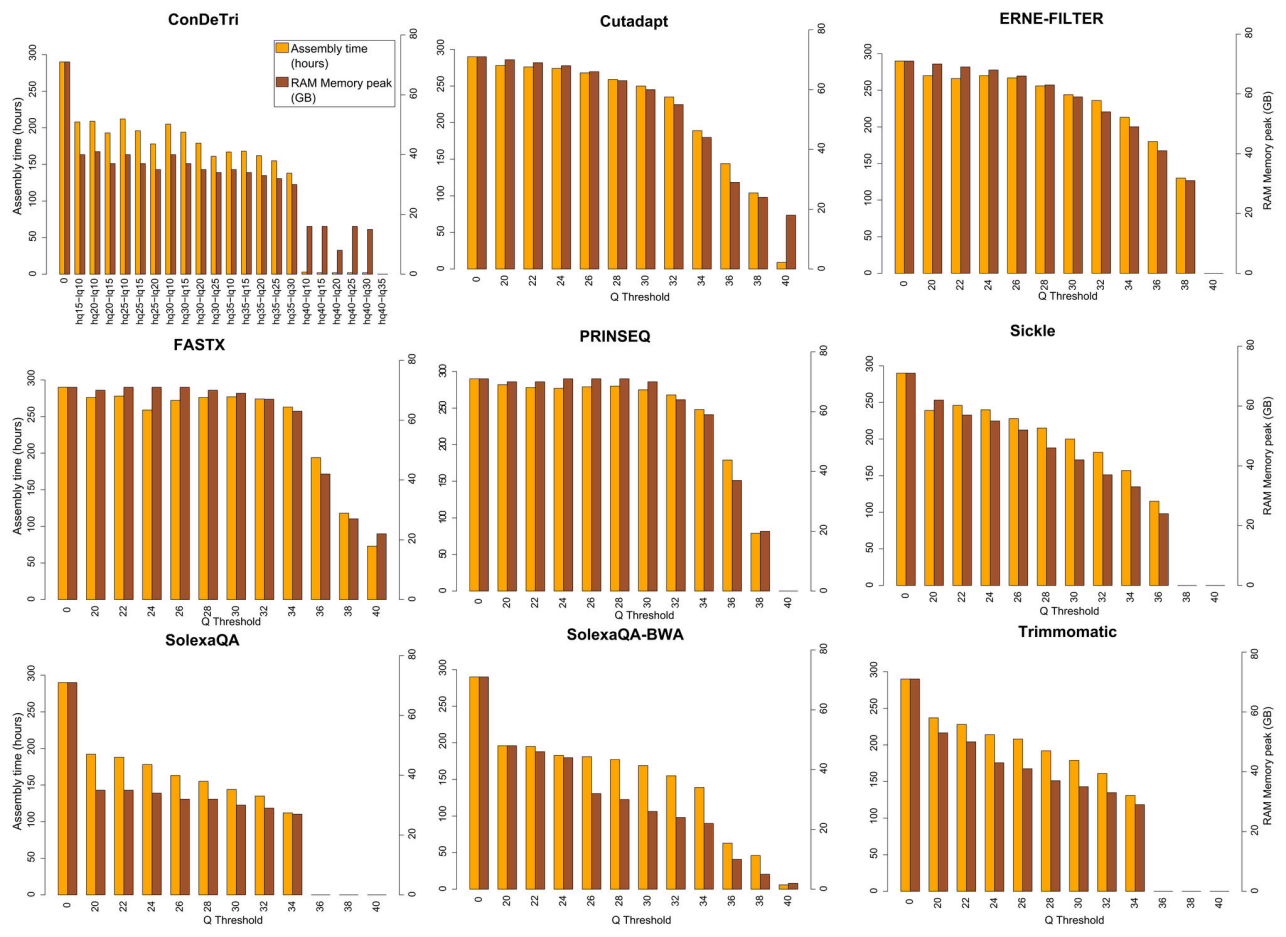
Computational requirements necessary for full *Prunus persica* genome assembly (RAM peak and time) for different combinations of read trimming tools and thresholds.

Read trimming affects only partially genome assembly results and there is no big difference among results from the different datasets (see Figure 5 and Table 2). Negative effects are seen for high quality values (e.g. Q>30) on most datasets. Trimmed datasets from ConDeTri, Trimmomatic, Sickle and especially SolexaQA produce slightly more fragmented assemblies and this is probably due to a more stringent trimming that reflects also on lower computational needs (see Figure 6). The assembler used, ABySS, models and deals with sequencing errors; therefore, assembly of the untrimmed dataset results best under certain metrics (average scaffold length, longest scaffold, N50 in bp) but at the cost of a slightly lower precision and a much higher computational demand. Conversely, stringent trimming tends to heavily remove data and decrease overall assembly quality.

### Overall effects of read trimming

An overall analysis of the three computational biology analyses investigated here allows us to draw three conclusions. First, trimming is beneficial in RNA-Seq, SNP identification and genome assembly procedures, with the best effects evident for intermediate quality thresholds (Q between 20 and 30). Second, while all tools behave quite well (compared to untrimmed scenarios), some datasets with specific issues or low overall quality (Figure 2) benefit more from the most recent algorithms that operate on both 5’ and 3’ ends of the read, such as ERNE-FILTER, or those allowing low quality islands surrounded by high quality stretches, such as ConDeTri. Third, the choice of an optimal threshold is always a tradeoff between the amount of information retained (i.e,. the number of surviving reads/nucleotides) and its reliability, i.e., in RNA-Seq the alignable fraction, in SNP identification the amount of true positive aligned nucleotides and in genome assembly the percentage of the scaffolds correctly assembled and mappable on the reference genome. Overall, trimming gives also an advantage in terms of computational resources used and execution time, assessed for genome assembly in the present study (Figure 6) but evident also for the other analyses (data not shown). The performance of trimming seems to be dependent on the Q ditribution of the input dataset. For example, we observe a sudden drop in called SNPs above Q trimming thresholds of around 35 (Figure 3); in facts, Q=35 is roughly the flection point in the Q distribution of the *Prunus persica* dataset (Figure S1). On the other hand, for the higher quality *Saccharomyces cerevisiae* dataset, the drop in called SNPs is indeed present, but more gradual, and observed at Q values above 36, while the Q distribution for this datasets shows a flection point at Q=37 (Figure S1).

## Discussion

It is clear that Next Generation Sequencing has become increasingly valuable in scientific research as well as in diagnostics. Data generated by this relatively novel approach, taking the form of billions of short nucleotide sequences, is the basis of contemporary Systems Biology and all studies that deal with the systemic complexity of cells and organisms. NGS gave researchers an unprecedented detail in studying the nature and the dynamics of nucleic acids, whether to obtain the continuous uninterrupted sequence of an organism genome or to quantify the entire population of mRNAs in a particular sample, aiming at the deeper understanding of expression-related molecular networks.

Despite an explosion in the usage of NGS data, not much literature deals with the best practices on how to treat short reads produced during an NGS experiment, before their usage in a particular biologically oriented analysis (e.g., genome assembly, gene expression analysis). In this paper, we investigated a much used but not well described step of quality preprocessing of NGS raw sequences, the trimming, which aims at keeping the highest possible information contained within reads, discarding low quality sequences or parts of sequences. Consequences of using untrimmed reads are reflected in missing or wrong alignments that negatively affect downstream analysis (e.g., transcriptome profiling, SNP, indel and Copy Number Variant detection). Furthermore, low quality untrimmed reads produce false k-mers that increase the chance of misassemblies and the amount of computational resources needed.

We described the underlying algorithms guiding the execution of nine amongst the most popular trimming programs currently available. We tested their relative performance on different publicly available datasets with different popular post-processing tasks in mind, namely genome assembly, gene expression analysis and SNP calling.

Our results show a non-obvious lack of overlap between different trimming programs, which is not immediately identifiable in the two different classes discernible by the algorithms’ formulas. Despite case by case scenario, we believe that our results show how trimming can increase the reliability of downstream analysis, while at the same time reducing computational requirements (RAM, disk space and execution time). However, different trimmers behave differently and are highly dependent on the parameters used; in the current paper, we explored the effects of the main quality parameter threshold Q, showing how a Q set too high can dramatically reduce the surviving dataset size, while a Q set too low keeps too many noisy or plainly random reads, decreasing dataset quality and unnecessarily increasing computational requirements. Therefore, it is up to the researcher to select the best tradeoff between read loss and dataset quality. In the specific case of RNA-Seq, the tradeoff between sensitivity (number of aligned reads) and specificity (number of correctly aligned reads) seems to be always detrimental when trimming the datasets (Figure S2); in such a case, the modern aligners, like Tophat, seem to be able to overcome low quality issues, therefore making trimming unnecessary.

As for the generic question “what is the best trimming algorithm?” no generic answer can be given, since this is highly dependent on the dataset, downstream analysis and user-decided parameter-dependent tradeoffs, as shown here. Moreover we show how 5 algorithms (Cutadapt, ERNE-FILTER, FASTX, PRINSEQ, and SolexaQA-BWA) exhibit a common behavior, while the other 4 algorithms (ConDeTri, Sickle, SolexaQA, and Trimmomatic) operate in more peculiar ways (see File S2). Indeed there is a correlation between the former trimmers and the results of the software that use the trimmed reads: the better results for de-novo assembly, SNP identification and gene-expressions derived from the window-based tools (see Materials and methods). However, when processing DNA-Seq data we can affirm that trimming should be applied every time in order to improve quality and performance; ideally, this should be done after a tuned and intelligent comparison of several trimmers and parameters. Also, as discussed in the Results, the general quality distribution for the dataset to analyze should be checked before the trimming process (Figure S1) in order to critically decide a specific Q threshold which includes most of the dataset. 

In conclusion, with this comprehensive comparison study on trimming procedures of NGS data, we hope to have reduced the combinatorial space for finding the ideal match between dataset/biological question and trimmer/parameters.

## Materials and Methods

### Trimming implementations

In the current paper we utilize a group of trimming programs, all of them being publicly available and open-source (Table 1).

The tools can be classified in two main classes, based on the algorithm type:

• Running sum algorithms (Cutadapt, ERNE-FILTER, and SolexaQA with -*bwa* option);
• Window based algorithms (ConDeTri, FASTX, PRINSEQ, Sickle, SolexaQA, and Trimmomatic).

In the following section we describe the general algorithms implemented in each of these trimming programs. The parameter Q is used in our comparative study as a variable threshold to test the trimming properties of all the programs, with the exception of ConDeTri, which requires two thresholds *hq* and *lq*. In our analysis, for all trimmers, if the length of the trimmed read drops below 70% of the original read [27], the read is entirely discarded.

### Running Sum Algorithms: Cutadapt

Cutadapt [32] implements the BWA trimming algorithm [9]. Given a quality threshold Q (option --*quality-cutoff*, default: none), a running sum *s* is calculated from the 3’-end to the 5’-end :

s(i)=s(i+1)+quality(i)−Q

where quality(*i*) is the PHRED quality of the *i*-th base. The formula for the 3’-end base (with *i* equaling the read length), is:

s(i)=quality(i)−Q

The position *i* where the running sum s(*i*) is *minimal* will be chosen as the position to cut-off the 3’ bad quality region. No base is trimmed at the 5’-end, unless if the number of the surviving bases goes below a minimum size specified by the parameter --*minimum-length*. In this case, the entire read is discarded.

### Running sum algorithms: SolexaQA (BWA implementation)

The BWA algorithm implemented in SolexaQA and accessed via the -*bwa* option [29] is basically identical to the one implemented in Cutadapt. The Q value must be specified by the –*h* option, with no default.

### Running sum algorithms: ERNE-FILTER

ERNE-FILTER (erne.sourceforge.net), proposed in the current manuscript, implements the PHRED/PHRAP modified Mott’s trimming algorithm (www.phrap.org/phredphrap/phred.html) and is described in the CLC workbench [33] user’s manual. This algorithm is similar to the BWA algorithm (implemented in Cutadapt and SolexaQA-bwa), though it runs in the reads rightwards rather than leftwards.

Given a threshold value Q (option --*min-phred-value-mott*, default: 20), the algorithm works in two steps. In the first step, it computes the first index *l* where the quality is greater than Q. In the second step, the program calculates S(*l*) = quality(*l*) - Q and the running sum:

S(i)=S(i−1)+quality(i)−Q

when *i* is greater than *l*. The part of the sequence not trimmed is the region between the position *l* and the last position whose running sum is maximal. Everything before and after is trimmed out.

After that, if the good region length is lower than a threshold (option --*min-size*, default: 25) or if the mean quality in the good region is lower than a threshold (option --*min-mean-phred-quality*, default: 15), then the read is discarded.

### Window based algorithms: ConDeTri

ConDeTri [16] operates, like BWA algorithm-based tools, from the 3’-end to the 5’-end of the read. It requires two quality threshold values: HQ (“high quality”, specified by the parameter -*hq*) and LQ (“low quality”, specified by the parameter -*lq*).

ConDeTri starts by removing all 3’-end bases with a score below HQ (default: 25). However, up to *ml* (default: 1) single bases with quality below HQ surrounded by high-quality bases can be kept temporarily, unless more than one consecutive base with quality below HQ is detected: in this situation, all previously kept sequence is trimmed. The trimming itself stop when a stretch of at least *mh* (default:5) consecutive bases is detected. The trimmed read is kept if at least a fraction of its bases (parameter *–frac*, default: 80%) has a quality score of at least HQ, with no base having quality below LQ (default: 10). The rationale is to dilute moderate error probability within long stretches of correct sequences.

### Window based algorithms: FASTX-Toolkit quality trimmer

FASTX-Toolkit (hannonlab.cshl.edu/fastx_toolkit) is a suite of programs for NGS data analysis. Its trimmer works as follows. Given a threshold Q (required option -*q*, no default) the algorithm searches within the whole sequence the longest continuous stretch of bases with associated qualities lower than Q. Then, all bases towards both ends of such a window are trimmed out.

### Window based algorithms: PRINSEQ

The algorithm implemented in PRINSEQ [30] is inherently identical to the one implemented in FASTX-Toolkit quality trimmer, with the exception that PRINSEQ can work from both ends of the read. However, PRINSEQ has several different options (around 30 classes) that can be tuned and combined with the basic trimming. For example, a fixed read fragment can be removed prior to the trimming itself, and special operations can be applied for dataset-specific tasks (e.g., it is possible to automatically remove polyA/polyT stretches from RNA-Seq reads). The main quality thresholds can be controlled independently for the two extremities: Q_left_ and Q_right_ (options --*trim_qual_left* and *--trim_qual_right*, no default values). In our study, we tested several thresholds with the constraint Q_left_ = Q_right_.

### Window based algorithms: Sickle

Sickle (github.com/najoshi/sickle) works with an adaptive window spanning the read from the 5'-end to the 3'-end, where the window's size is 10% of the whole read length. Given a quality threshold Q (option -q, default: 20), Sickle searches the starting position *i* where the average quality of the bases in the window is greater than or equal to Q: the first *i-*1 bases are trimmed out. Then the window continues to slide to the 3'-end until the average quality is greater or equal to Q. As soon as the average quality drops below Q, the program identifies the cut position to trim out bases from the 3'-end. 

### Window based algorithms: SolexaQA (Dynamic Trim implementation)

The default algorithm implemented by SolexaQA was named “Dynamic Trim” [29]. This method accepts an error probability p (using option -*p*) or a quality value Q (using option -*h*). If no user-specified parameters are passed, it uses the error probability with a default of p=0.05 (equivalent to quality score Q≈13). Given a quality value Q (or obtained by converting p using (1)), the algorithm computes the longest contiguous stretch of bases whose quality values are all greater than Q and discards all other bases.

### Window based algorithms: Trimmomatic

Trimmomatic [34] works with a user-defined window spanning the read from 5' to 3' and removes bases only at 3'-end. Given a window's length and a quality threshold Q (the option *SLIDINGWINDOW* takes two parameters and it has no default values), the algorithm cuts the 3'-end when the average quality drops below Q.

All trimming tools were run on GNU/Linux machines at varying Q thresholds (single Q for most, with Q_left_ = Q_right_ for PRINSEQ, combinations of HQ and LQ for ConDeTri). Example usages for all tools are provided in File S3. 

### Datasets

Two distinct RNA-Seq samples were obtained from the Sequence Read Archive (SRA) [35]. The first one is composed by short Illumina reads (33 bp) derived from the *Homo sapiens* ENCODE cell line HepG2 [36]. This cell line is characterized by a relatively normal karyotype and is derived from liver carcinoma (SRA entry SRR002073). The second sample is derived from untreated *Arabidopsis thaliana* roots, and is composed by longer reads (83 bp, SRA entry SRR420813).

For SNP identification and genome assembly tests we used two different DNA-Seq sample sets. The first, obtained from a single diploid inbred *Prunus persica* individual (the same used for Sanger-based sequencing of the reference genome), is available as SRA accession number SRX150254 [37], and is constituted by 100 bp paired-end Illumina reads. The second DNA-Seq sample contains 100 bp paired-end Illumina reads from the *Saccharomyces cerevisiae* strain YDJ25, which is nearly isogenic to the yeast strain used to build the reference genome (S288C). The yeast reads were generated within a large screening study of several yeast strains and it is available at SRA entry SRR452441 [38].

FastQC [39] quality assessments for all these samples are available in File S1.

### Post-Processing Quality Assessments: Gene expression analysis

Human reads were aligned to the *Homo sapiens* genome sequence (hg19 v2) using Tophat v2.0.5 with default parameters [40] under the guidance of the latest (downloaded 8-April-2013) hg19 UCSC gene annotation [41]. *Arabidopsis thaliana* reads were aligned using the same tool with the latest TAIR10 genome and gene annotation [42]. Numbers of mapped and unmapped reads and nucleotides were obtained analyzing the output alignment files generated by Tophat.

### Post-Processing Quality Assessments: De novo genome assembly

Raw and trimmed yeast and peach datasets were *de novo* assembled with the parallel implementation of ABySS version 1.3.4 [19] with default parameters except: k=71, b=1000, p=0.95 and s=500, in order to reliably maximize assembly and fuse possible heterozygous regions in a single sequence [37]. Genome assembly was split in two phases in order to run the first distributed phase of ABySS in a cluster using 8 nodes with 8 CPUs each and the second multi-threaded phase on a single node with 8 CPUs. Running CPU time is the sum of all CPUs time over the two phases and memory peak is the peak of memory usage during the first phase (Table S1). Total scaffold assembly size, number of sequences, average and maximum length, N50 and L50 were computed for each assembly with the GAM suite [43] and are reported in Table S1.

Scaffolds of each assembly were aligned to the corresponding reference genome using NUCmer, part of the software package MUMmer version 3.23 [44], with anchor matches that are unique in both the reference and query (*--mum*). Query sequences that aligned to the reference genome at minimum 95% identity over at least 90% of query length were extracted using show-tiling, still part of MUMmer, with default parameters but *–i 95 –v 90 –V 0* –*a –c* –*g -1*. Such fragments represent a tiling path of queries and their sum was defined “Assembly tiled” in (Table S1). The proportion of assembly tiled over the whole assembly or the reference genome represents, respectively, precision and recall.

The *Prunus persica* genome v1.0 was used as reference in this paper, and is available at GenBank under the accession number AKXU01000000 [37]. The reference *Saccharomyces cerevisiae* genome (release R64) was obtained from the Saccharomyces Genome Database [45].

### Post-Processing Quality Assessments: SNP identification

Yeast and peach raw and trimmed reads were aligned over the original genomes using BWA version 0.6.2 [9]. Single Nucleotide Polymorphisms were then detected and counted using VarScan v2.3.3 [11] with loose parameters (*--min-coverage 1 --min-reads2 1 --min-var-freq 0.01 --min-avg-qual 0*). After the loose SNP call, Average Percentage Of Minor Allele Calls (APOMAC) was calculated as the total number of non-main allele nucleotides divided by the total number of aligned nucleotides. Average Percentage Of Non-reference Allele Calls (APONAC), which by definition is always greater than or equal to APOMAC, was calculated as the total number of non-reference allele nucleotides divided by the total number of aligned nucleotides. SNPs were called via the internal VarScan statistical framework with a p-value threshold of 0.05. An additional filter is applied to these SNPs: they must fall within nucleotide positions with coverage ranging between 0.5x and 1.5x of the average coverage on the genome (calculated every time for each dataset/trimmer/threshold combination) and must have a minimum minor allele frequency (maf) of at least 30%.

## Supporting Information

Figure S1 **Distributions of Q scores in the datasets assessed in this study.** Plots were generated using FastQC (http://www.bioinformatics.babraham.ac.uk/projects/fastqc).
(TIF)

Figure S2 **Barplots indicating the percentage of reads mapping within annotated UCSC gene models for the human RNA-Seq dataset SRR002073.** Three Q thresholds are tested for each trimming tool; for ConDeTri, Q corresponds to HQ and the LQ level was set to 10. The black line indicates the total number of aligned reads at Q=25 (in Millions).
(TIFF)

File S1 **FastQC-generated quality plots for the datasets analyzed in this study.**
(ZIP)

File S2 **Comparative analysis of the overlaps of the trimming tools investigated in the current paper.**
(DOCX)

File S3 **Collection of scripts and example pipelines to run the trimming tools.**
(ZIP)

Table S1 **Complete results of the trimming analysis performed in this study.** Sheet 1-4 contains the results for the four datasets: *Prunus persica, Saccharomyces cerevisiae, Homo sapiens* and *Arabidopsis thaliana*. Sheet 5 contains information on the total amount of aligned reads for the human RNA-Seq experiment. Sheet 6 contains the results of the coverage analysis used to generate Figure 4.
(XLSX)
