# Supplementary figures and images for: An Extensive Evaluation of Read Trimming Effects on Illumina NGS Data Analysis
Source: PLoS One. 2013 Dec 23;8(12):e85024. doi: 10.1371/journal.pone.0085024 (PMC3871669; doi:10.1371/journal.pone.0085024)

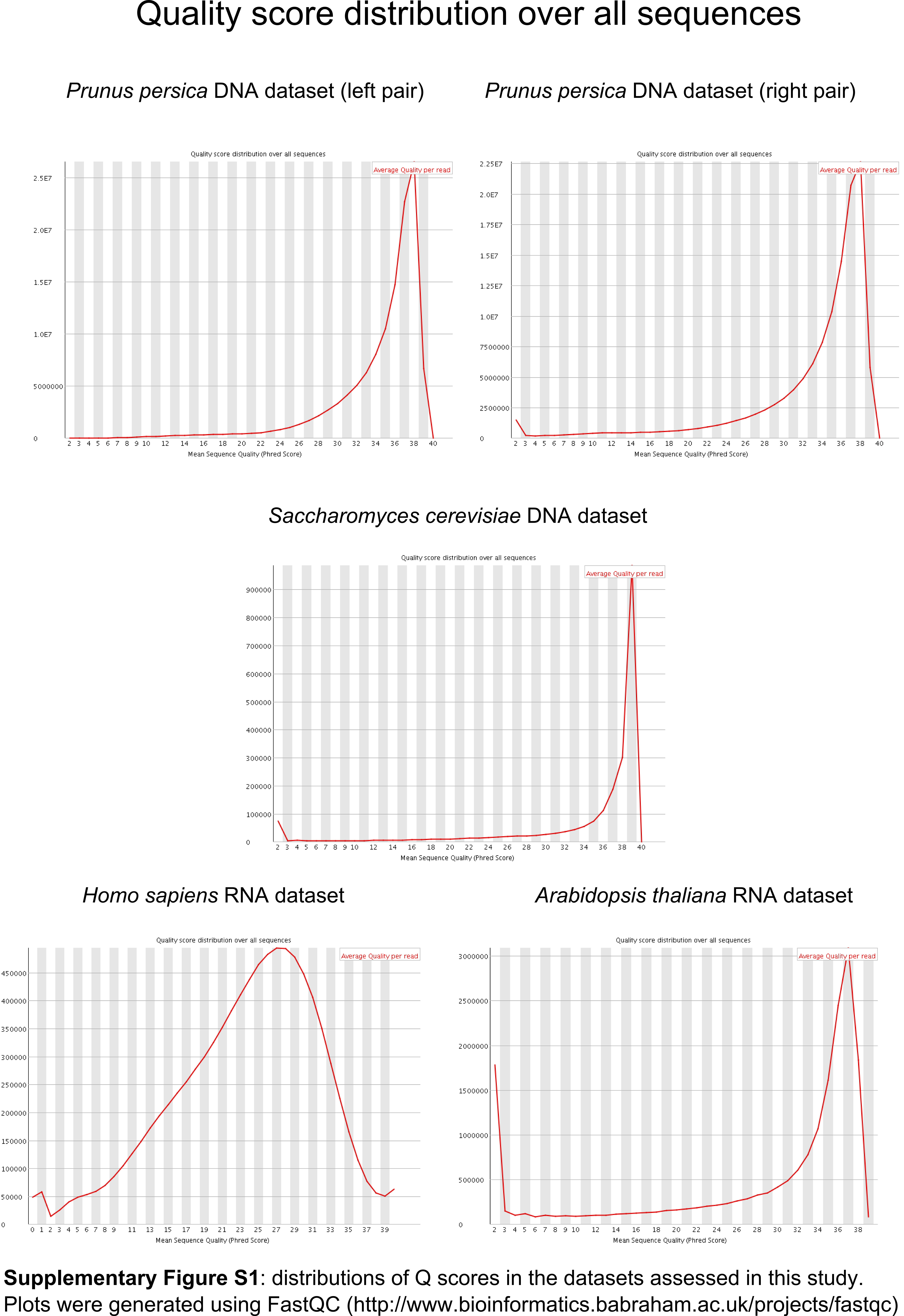

Supplement: Figure S1 — Distributions of Q scores in the datasets assessed in this study. Plots were generated using FastQC (http://www.bioinformatics.babraham.ac.uk/projects/fastqc). (TIF) [file pone.0085024.s001.tif]

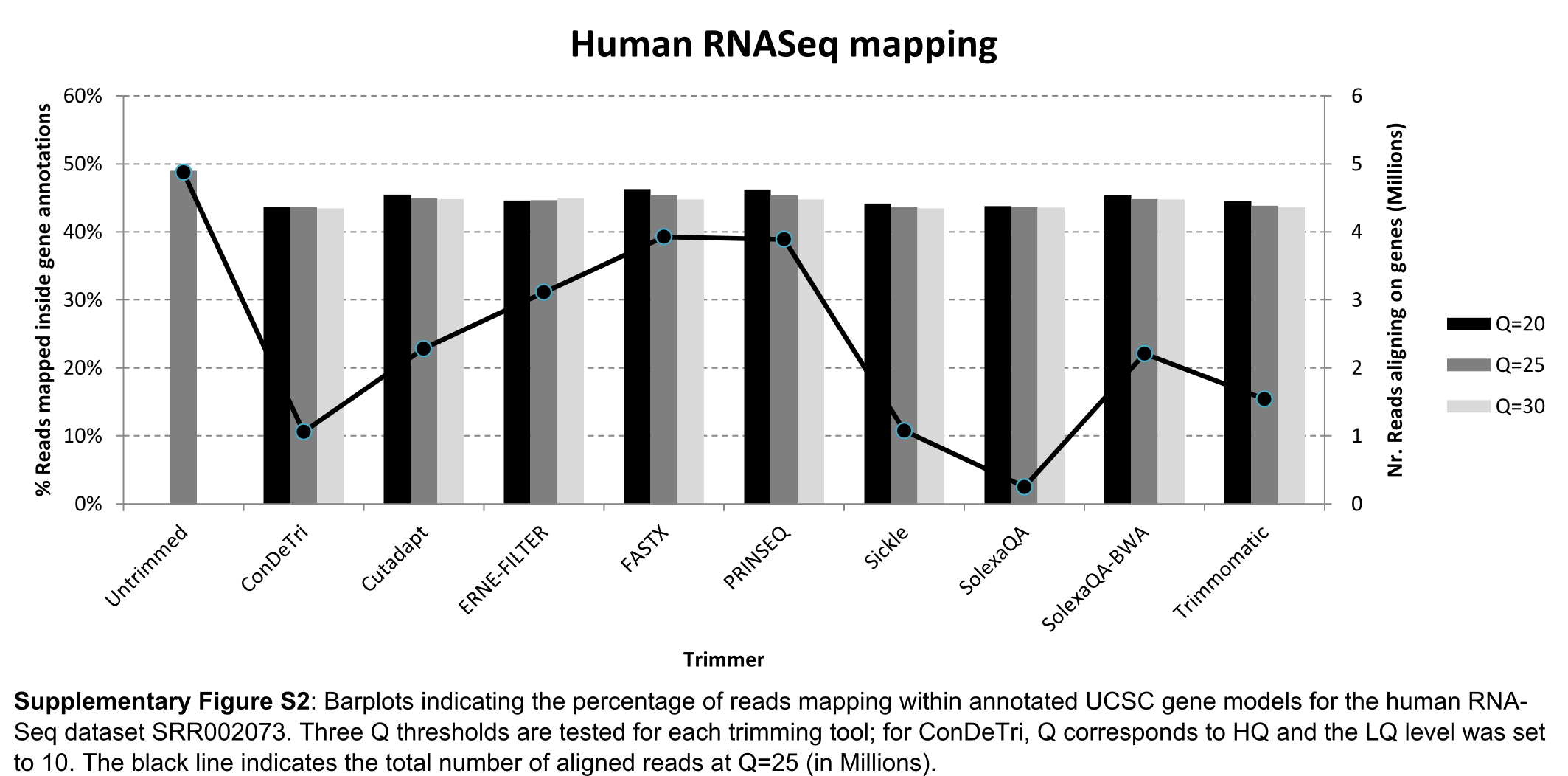

Supplement: Figure S2 — Barplots indicating the percentage of reads mapping within annotated UCSC gene models for the human RNA-Seq dataset SRR002073. Three Q thresholds are tested for each trimming tool; for ConDeTri, Q corresponds to HQ and the LQ level was set to 10. The black line indicates the total number of aligned reads at Q=25 (in Millions). (TIFF) [file pone.0085024.s002.tiff]

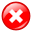

Supplement: File S1 — FastQC-generated quality plots for the datasets analyzed in this study. (ZIP) [file pone.0085024.s003.zip › fastqc/atrnaseq_SRR420813_1_fastqc/Icons/error.png]

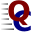

Supplement: File S1 — FastQC-generated quality plots for the datasets analyzed in this study. (ZIP) [file pone.0085024.s003.zip › fastqc/atrnaseq_SRR420813_1_fastqc/Icons/fastqc_icon.png]

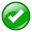

Supplement: File S1 — FastQC-generated quality plots for the datasets analyzed in this study. (ZIP) [file pone.0085024.s003.zip › fastqc/atrnaseq_SRR420813_1_fastqc/Icons/tick.png]

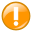

Supplement: File S1 — FastQC-generated quality plots for the datasets analyzed in this study. (ZIP) [file pone.0085024.s003.zip › fastqc/atrnaseq_SRR420813_1_fastqc/Icons/warning.png]

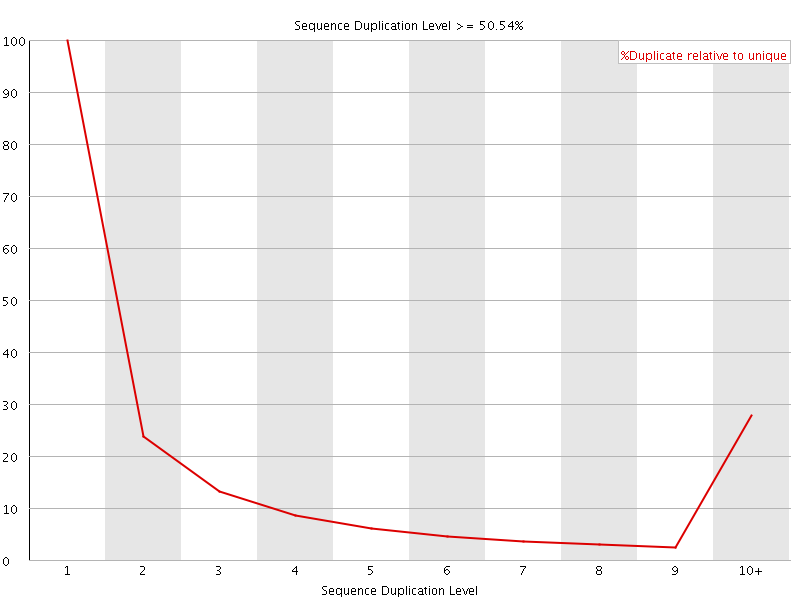

Supplement: File S1 — FastQC-generated quality plots for the datasets analyzed in this study. (ZIP) [file pone.0085024.s003.zip › fastqc/atrnaseq_SRR420813_1_fastqc/Images/duplication_levels.png]

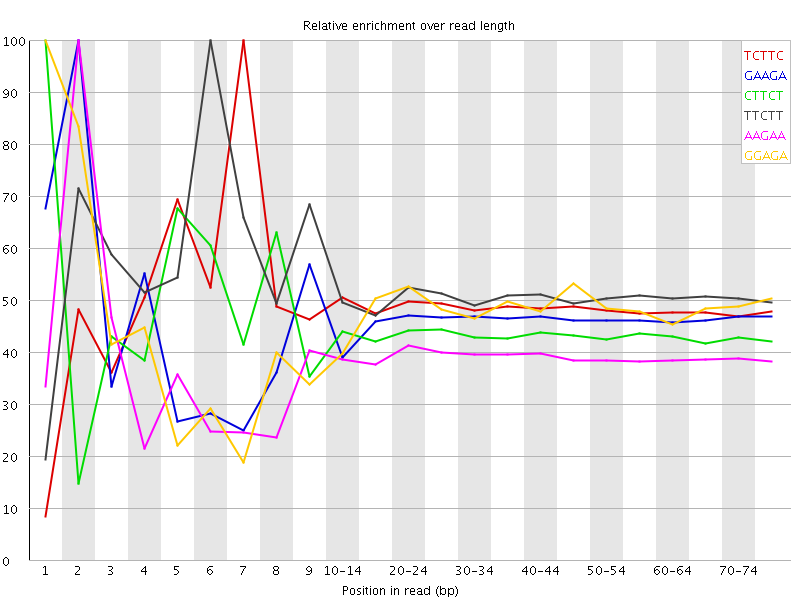

Supplement: File S1 — FastQC-generated quality plots for the datasets analyzed in this study. (ZIP) [file pone.0085024.s003.zip › fastqc/atrnaseq_SRR420813_1_fastqc/Images/kmer_profiles.png]

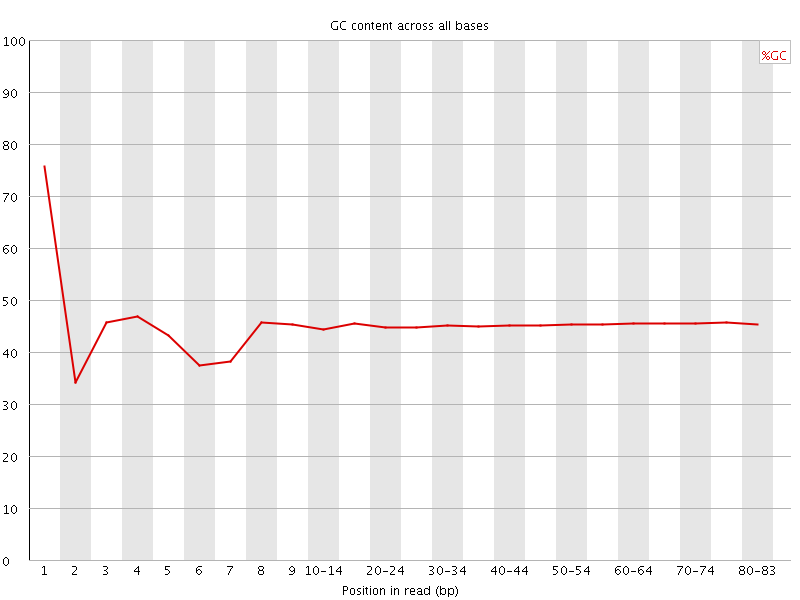

Supplement: File S1 — FastQC-generated quality plots for the datasets analyzed in this study. (ZIP) [file pone.0085024.s003.zip › fastqc/atrnaseq_SRR420813_1_fastqc/Images/per_base_gc_content.png]

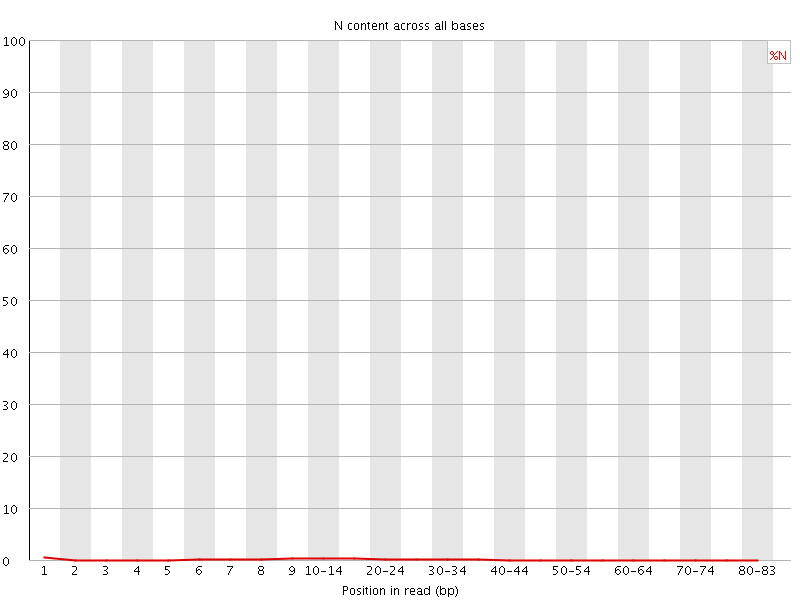

Supplement: File S1 — FastQC-generated quality plots for the datasets analyzed in this study. (ZIP) [file pone.0085024.s003.zip › fastqc/atrnaseq_SRR420813_1_fastqc/Images/per_base_n_content.png]

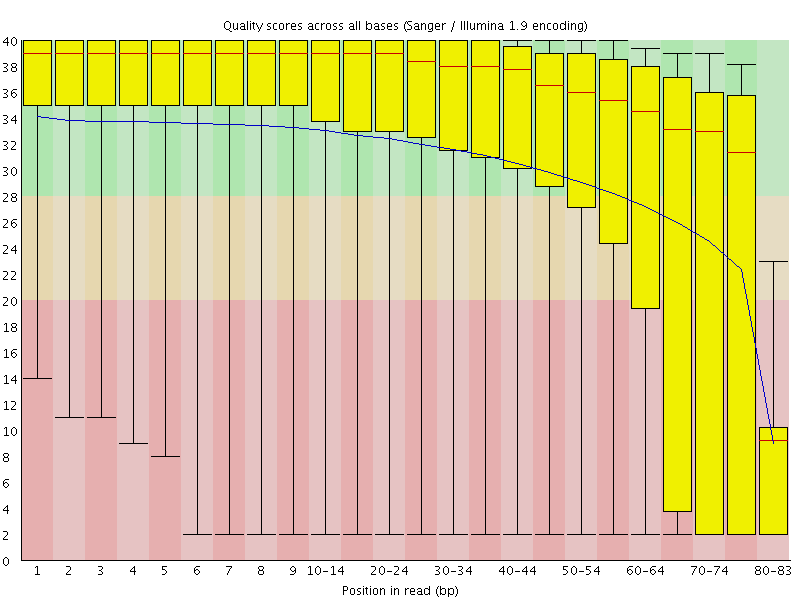

Supplement: File S1 — FastQC-generated quality plots for the datasets analyzed in this study. (ZIP) [file pone.0085024.s003.zip › fastqc/atrnaseq_SRR420813_1_fastqc/Images/per_base_quality.png]

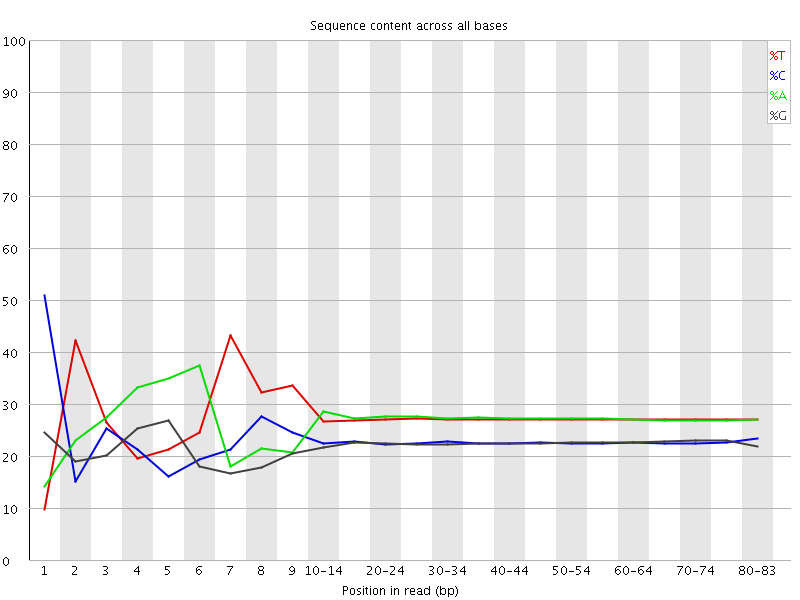

Supplement: File S1 — FastQC-generated quality plots for the datasets analyzed in this study. (ZIP) [file pone.0085024.s003.zip › fastqc/atrnaseq_SRR420813_1_fastqc/Images/per_base_sequence_content.png]

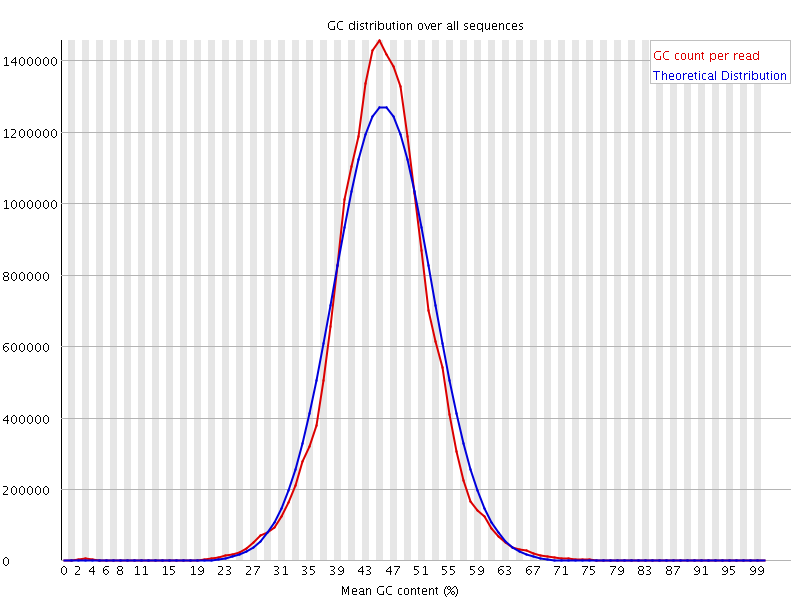

Supplement: File S1 — FastQC-generated quality plots for the datasets analyzed in this study. (ZIP) [file pone.0085024.s003.zip › fastqc/atrnaseq_SRR420813_1_fastqc/Images/per_sequence_gc_content.png]

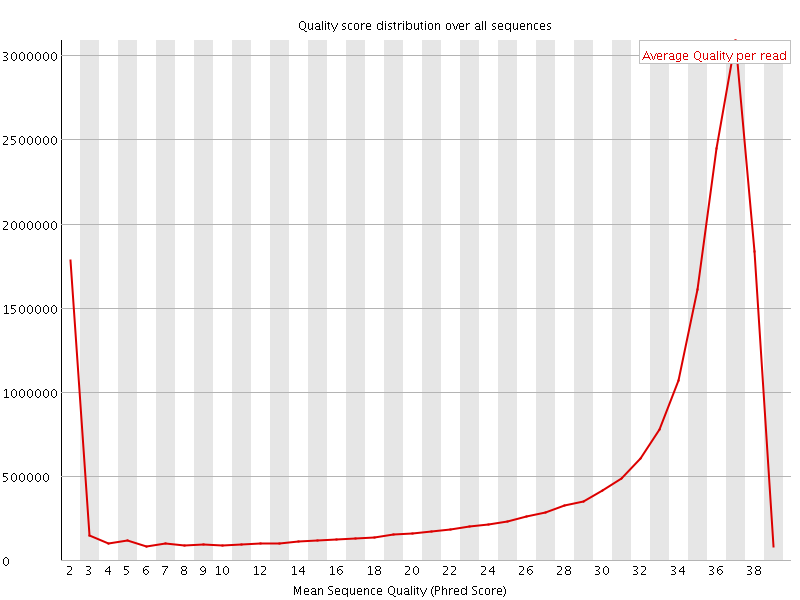

Supplement: File S1 — FastQC-generated quality plots for the datasets analyzed in this study. (ZIP) [file pone.0085024.s003.zip › fastqc/atrnaseq_SRR420813_1_fastqc/Images/per_sequence_quality.png]

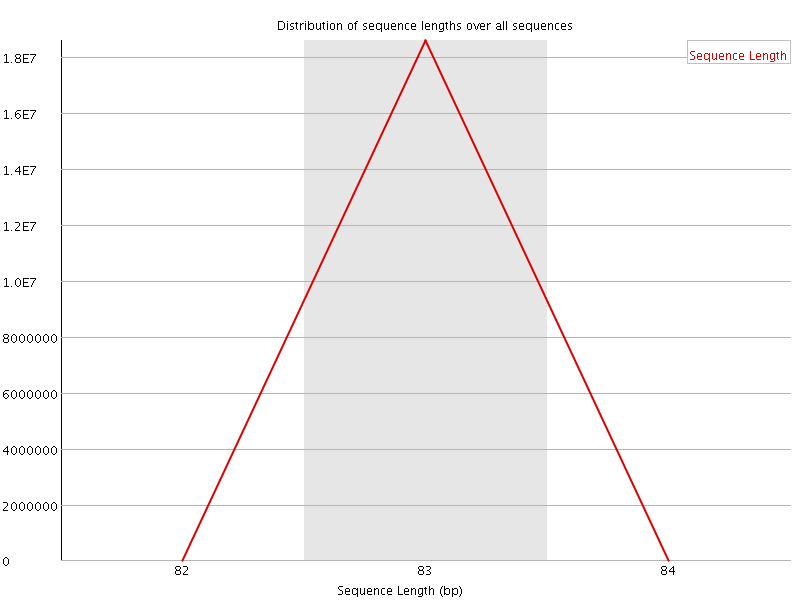

Supplement: File S1 — FastQC-generated quality plots for the datasets analyzed in this study. (ZIP) [file pone.0085024.s003.zip › fastqc/atrnaseq_SRR420813_1_fastqc/Images/sequence_length_distribution.png]

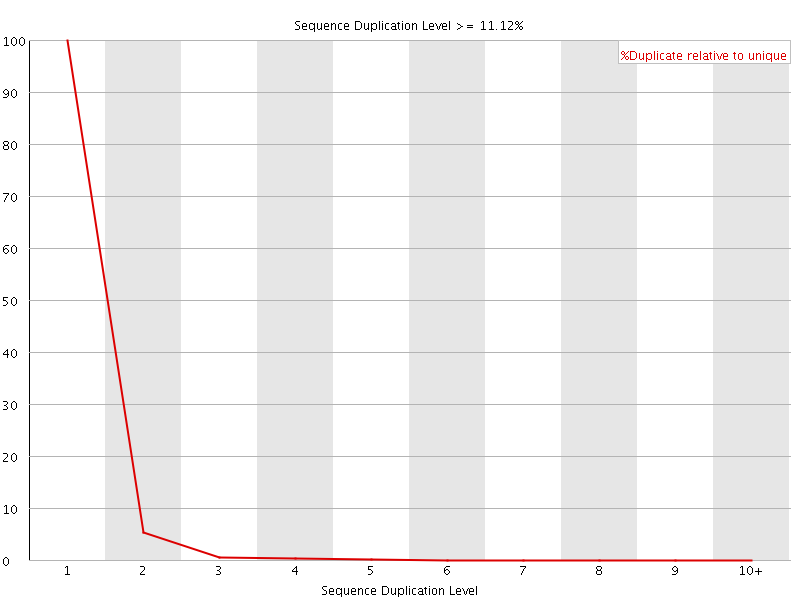

Supplement: File S1 — FastQC-generated quality plots for the datasets analyzed in this study. (ZIP) [file pone.0085024.s003.zip › fastqc/genotyping_yeast_SRR452441_1_fastqc/Images/duplication_levels.png]

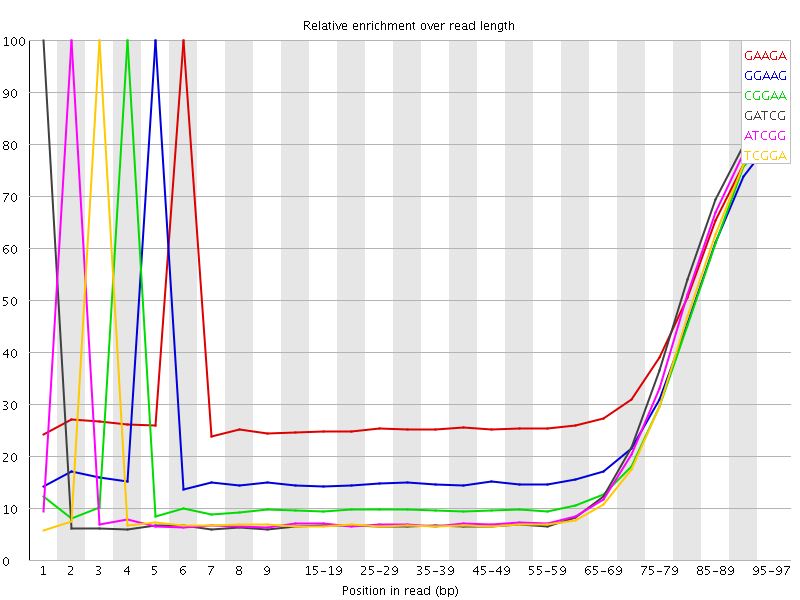

Supplement: File S1 — FastQC-generated quality plots for the datasets analyzed in this study. (ZIP) [file pone.0085024.s003.zip › fastqc/genotyping_yeast_SRR452441_1_fastqc/Images/kmer_profiles.png]

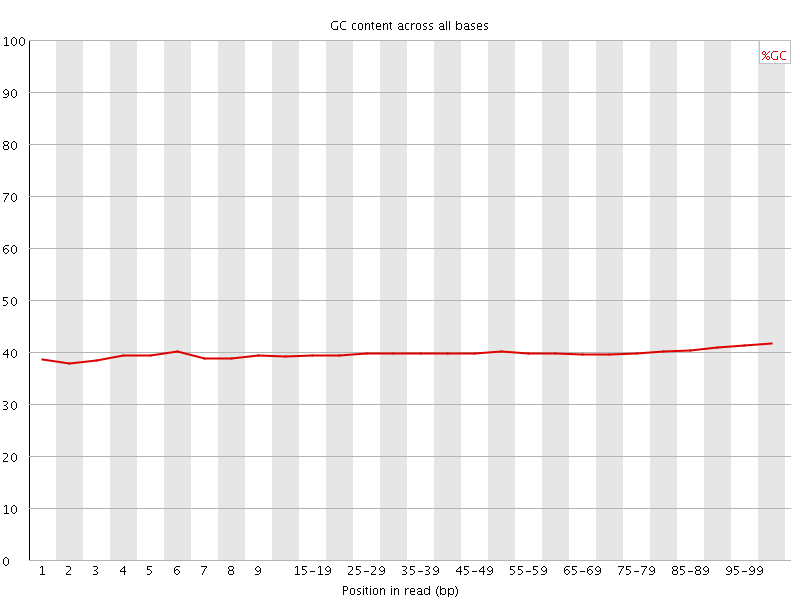

Supplement: File S1 — FastQC-generated quality plots for the datasets analyzed in this study. (ZIP) [file pone.0085024.s003.zip › fastqc/genotyping_yeast_SRR452441_1_fastqc/Images/per_base_gc_content.png]

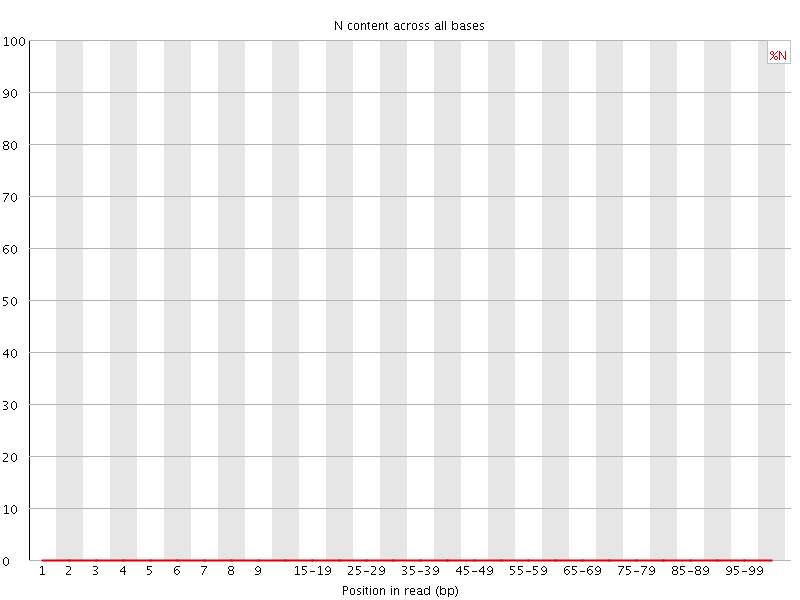

Supplement: File S1 — FastQC-generated quality plots for the datasets analyzed in this study. (ZIP) [file pone.0085024.s003.zip › fastqc/genotyping_yeast_SRR452441_1_fastqc/Images/per_base_n_content.png]

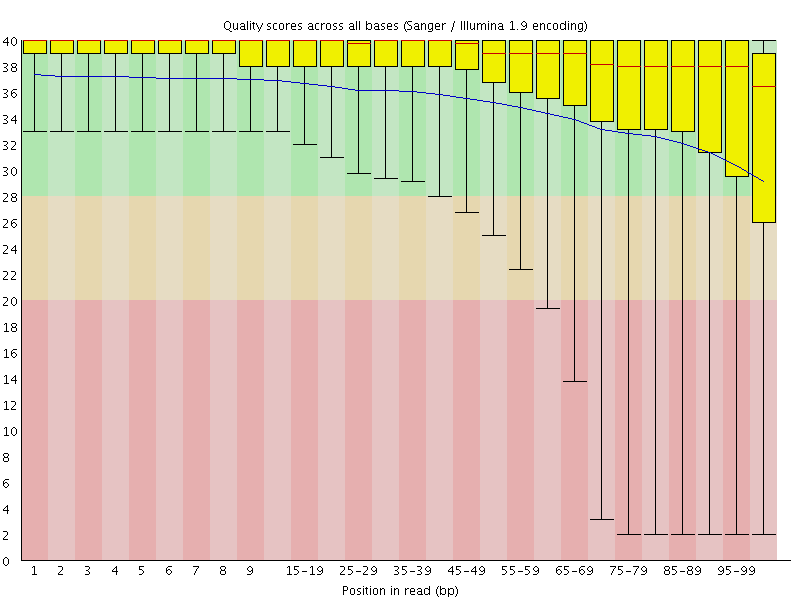

Supplement: File S1 — FastQC-generated quality plots for the datasets analyzed in this study. (ZIP) [file pone.0085024.s003.zip › fastqc/genotyping_yeast_SRR452441_1_fastqc/Images/per_base_quality.png]

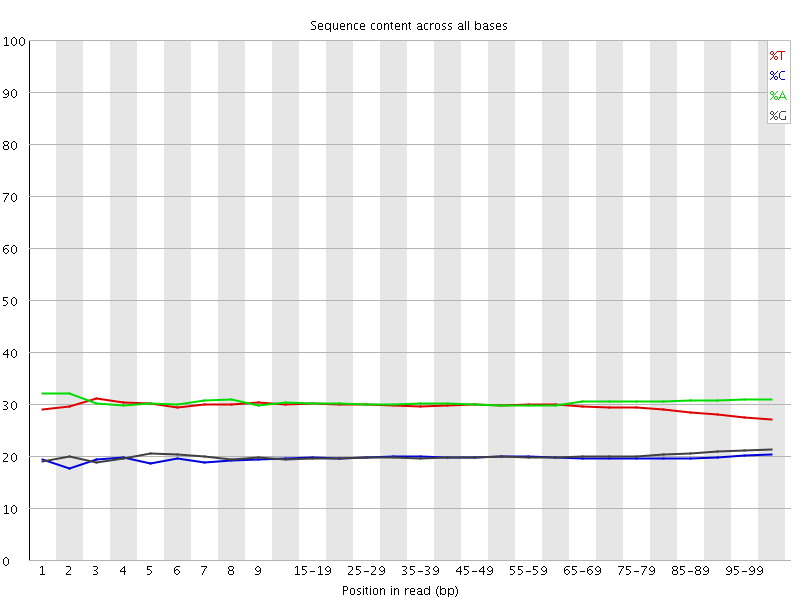

Supplement: File S1 — FastQC-generated quality plots for the datasets analyzed in this study. (ZIP) [file pone.0085024.s003.zip › fastqc/genotyping_yeast_SRR452441_1_fastqc/Images/per_base_sequence_content.png]

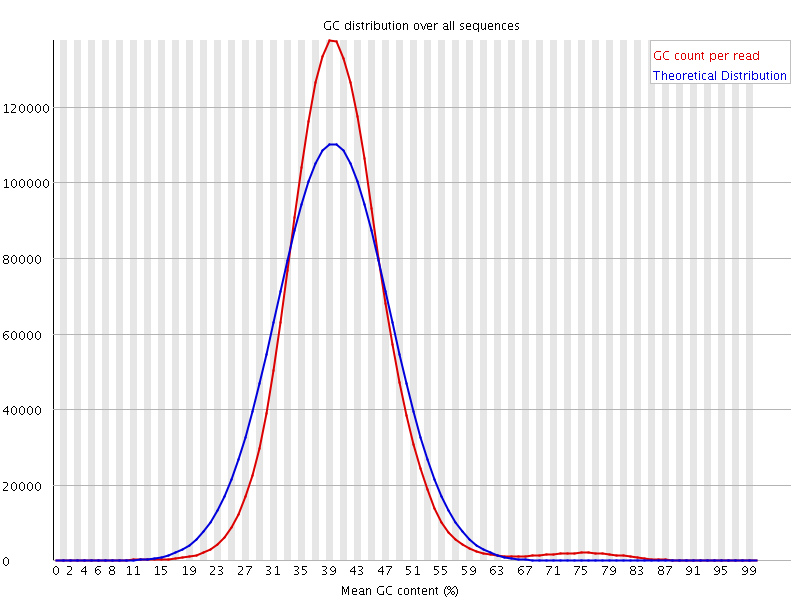

Supplement: File S1 — FastQC-generated quality plots for the datasets analyzed in this study. (ZIP) [file pone.0085024.s003.zip › fastqc/genotyping_yeast_SRR452441_1_fastqc/Images/per_sequence_gc_content.png]

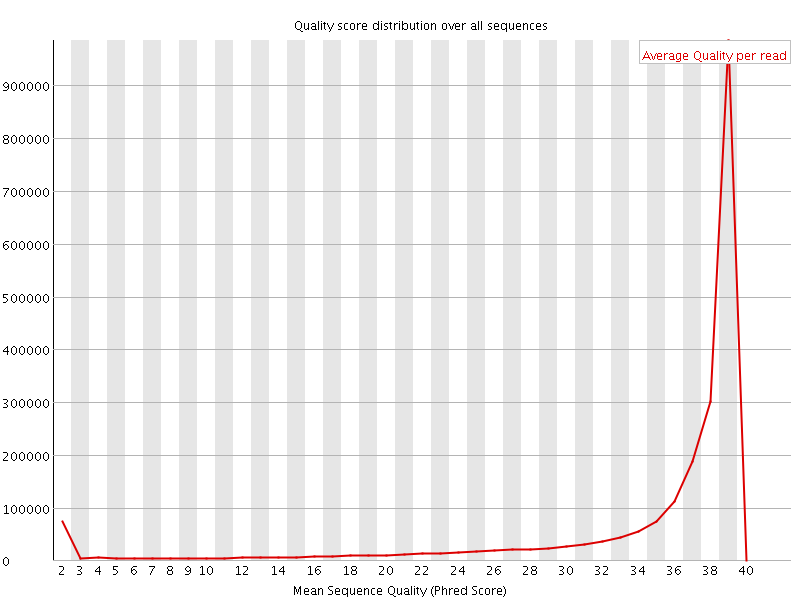

Supplement: File S1 — FastQC-generated quality plots for the datasets analyzed in this study. (ZIP) [file pone.0085024.s003.zip › fastqc/genotyping_yeast_SRR452441_1_fastqc/Images/per_sequence_quality.png]

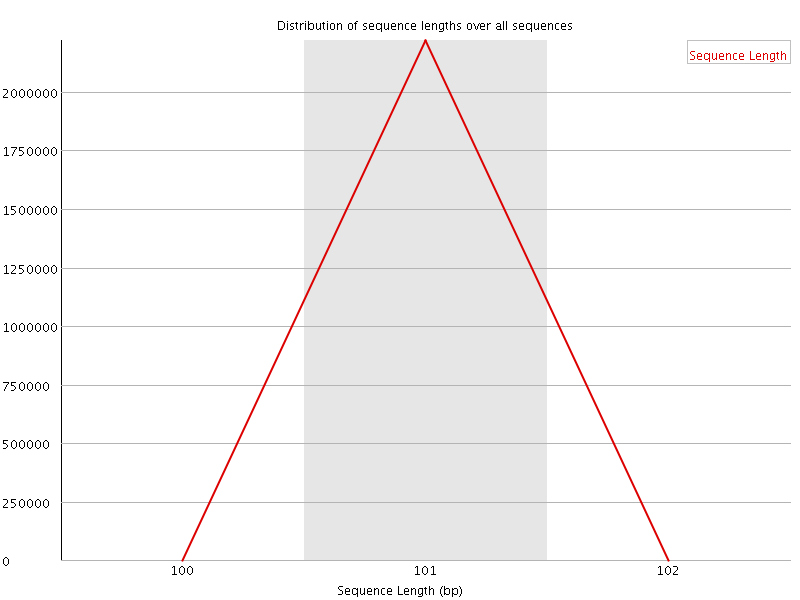

Supplement: File S1 — FastQC-generated quality plots for the datasets analyzed in this study. (ZIP) [file pone.0085024.s003.zip › fastqc/genotyping_yeast_SRR452441_1_fastqc/Images/sequence_length_distribution.png]

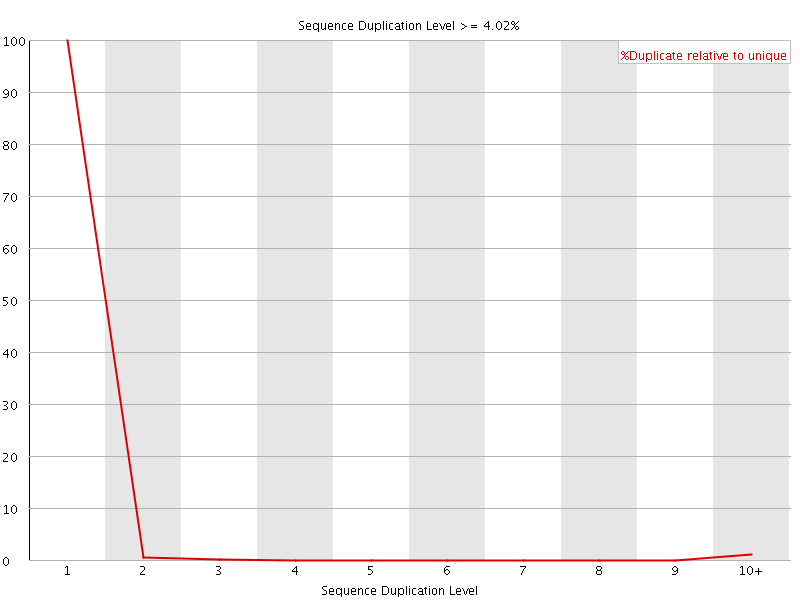

Supplement: File S1 — FastQC-generated quality plots for the datasets analyzed in this study. (ZIP) [file pone.0085024.s003.zip › fastqc/hsrnaseq_SRR002073_1_fastqc/Images/duplication_levels.png]

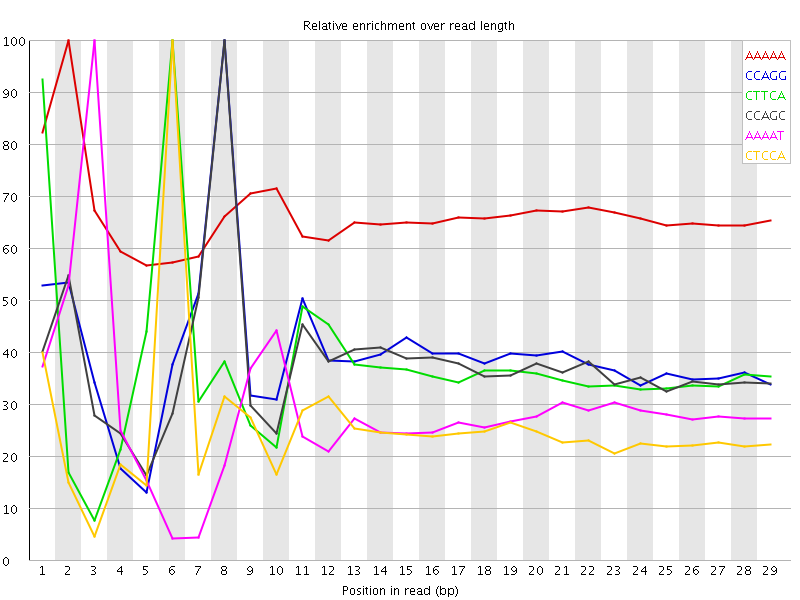

Supplement: File S1 — FastQC-generated quality plots for the datasets analyzed in this study. (ZIP) [file pone.0085024.s003.zip › fastqc/hsrnaseq_SRR002073_1_fastqc/Images/kmer_profiles.png]

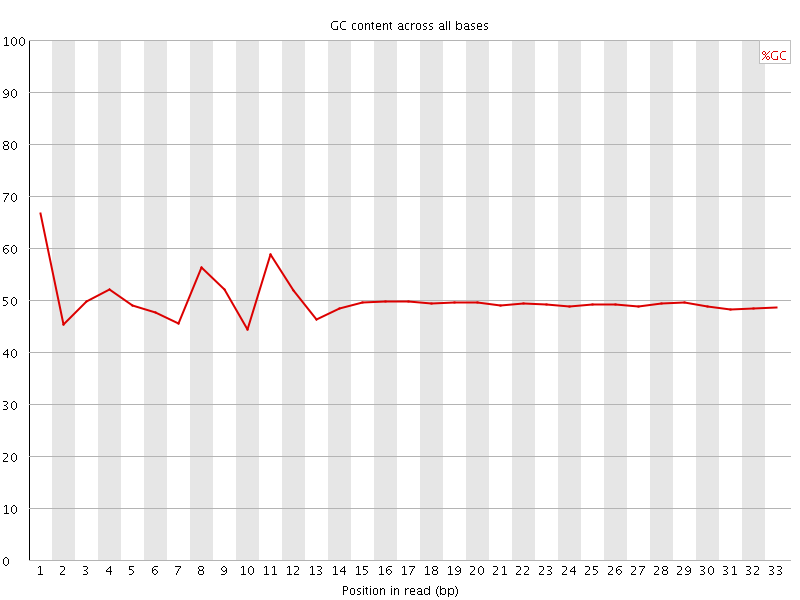

Supplement: File S1 — FastQC-generated quality plots for the datasets analyzed in this study. (ZIP) [file pone.0085024.s003.zip › fastqc/hsrnaseq_SRR002073_1_fastqc/Images/per_base_gc_content.png]

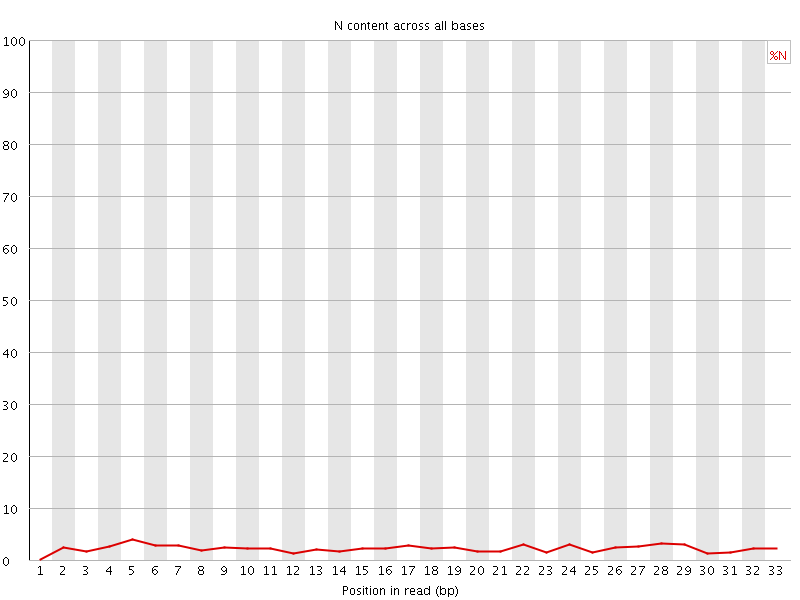

Supplement: File S1 — FastQC-generated quality plots for the datasets analyzed in this study. (ZIP) [file pone.0085024.s003.zip › fastqc/hsrnaseq_SRR002073_1_fastqc/Images/per_base_n_content.png]

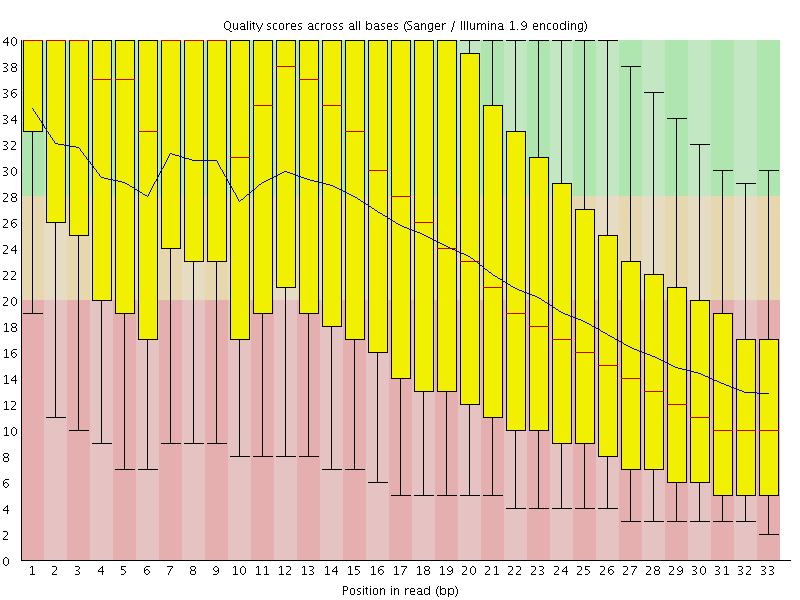

Supplement: File S1 — FastQC-generated quality plots for the datasets analyzed in this study. (ZIP) [file pone.0085024.s003.zip › fastqc/hsrnaseq_SRR002073_1_fastqc/Images/per_base_quality.png]

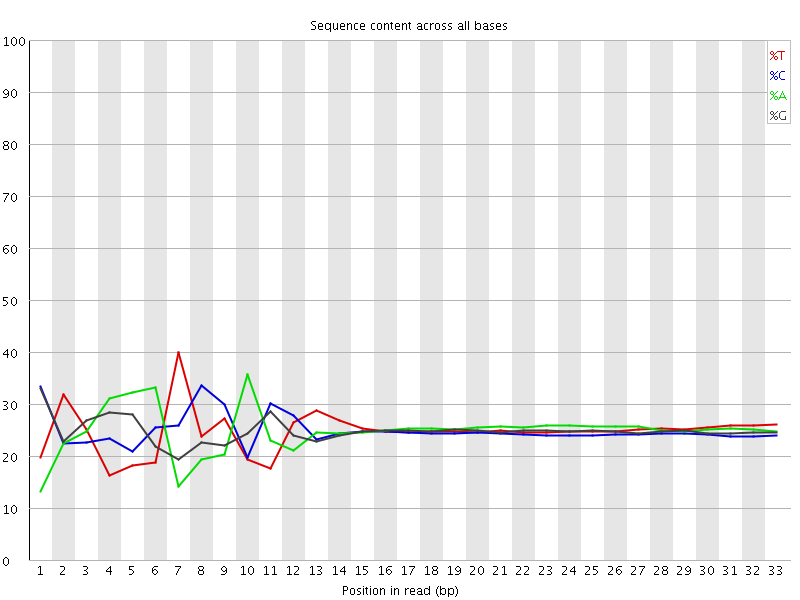

Supplement: File S1 — FastQC-generated quality plots for the datasets analyzed in this study. (ZIP) [file pone.0085024.s003.zip › fastqc/hsrnaseq_SRR002073_1_fastqc/Images/per_base_sequence_content.png]

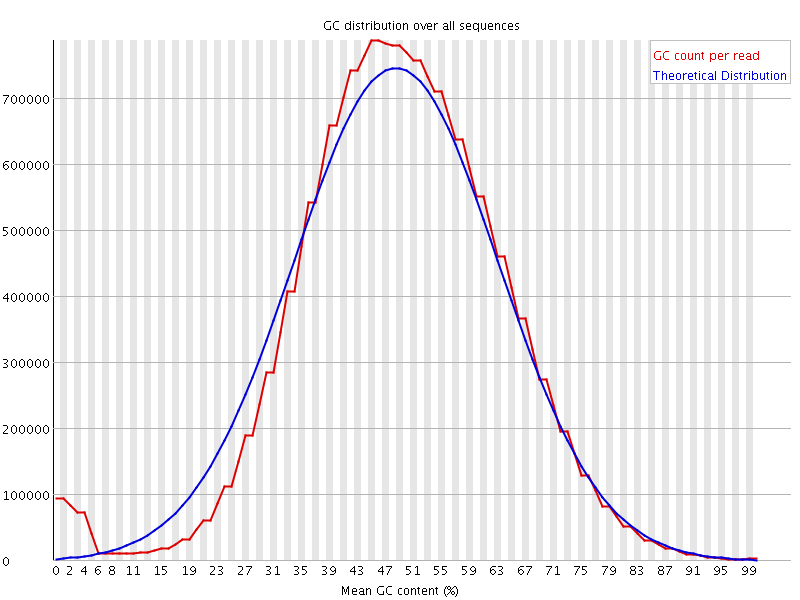

Supplement: File S1 — FastQC-generated quality plots for the datasets analyzed in this study. (ZIP) [file pone.0085024.s003.zip › fastqc/hsrnaseq_SRR002073_1_fastqc/Images/per_sequence_gc_content.png]

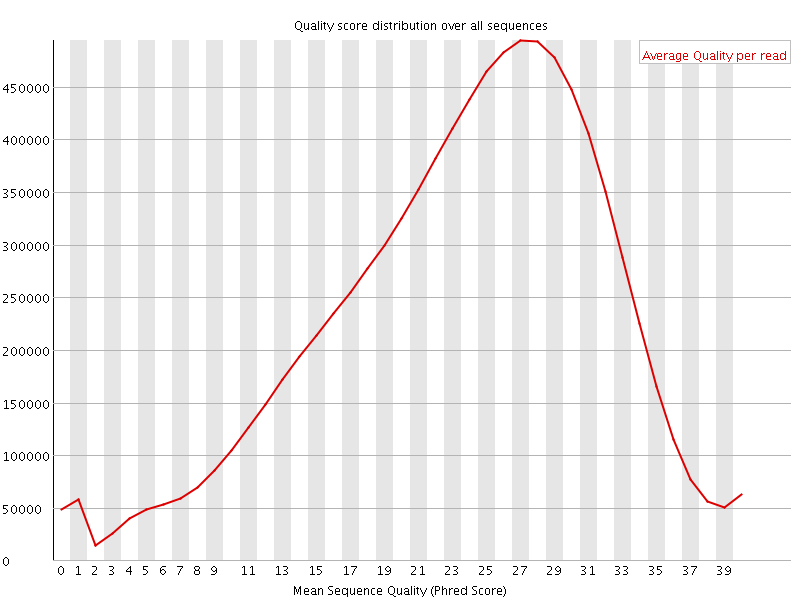

Supplement: File S1 — FastQC-generated quality plots for the datasets analyzed in this study. (ZIP) [file pone.0085024.s003.zip › fastqc/hsrnaseq_SRR002073_1_fastqc/Images/per_sequence_quality.png]

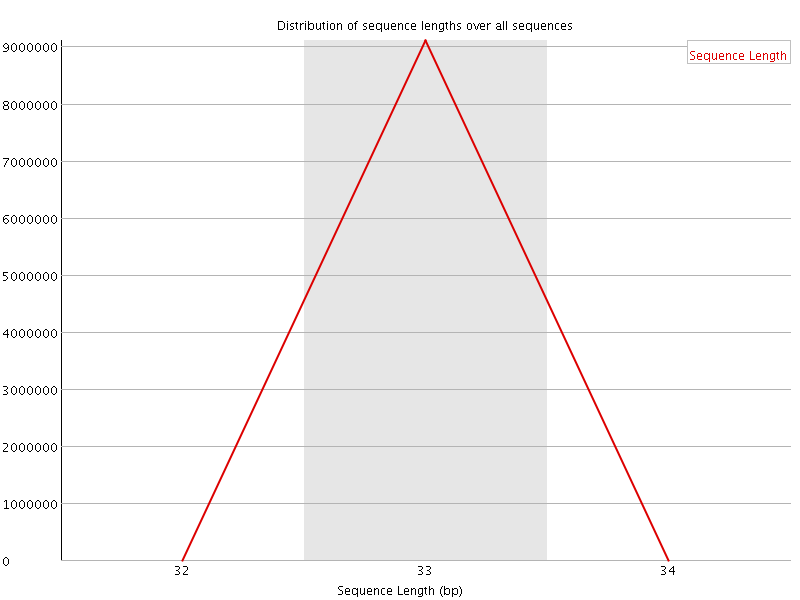

Supplement: File S1 — FastQC-generated quality plots for the datasets analyzed in this study. (ZIP) [file pone.0085024.s003.zip › fastqc/hsrnaseq_SRR002073_1_fastqc/Images/sequence_length_distribution.png]

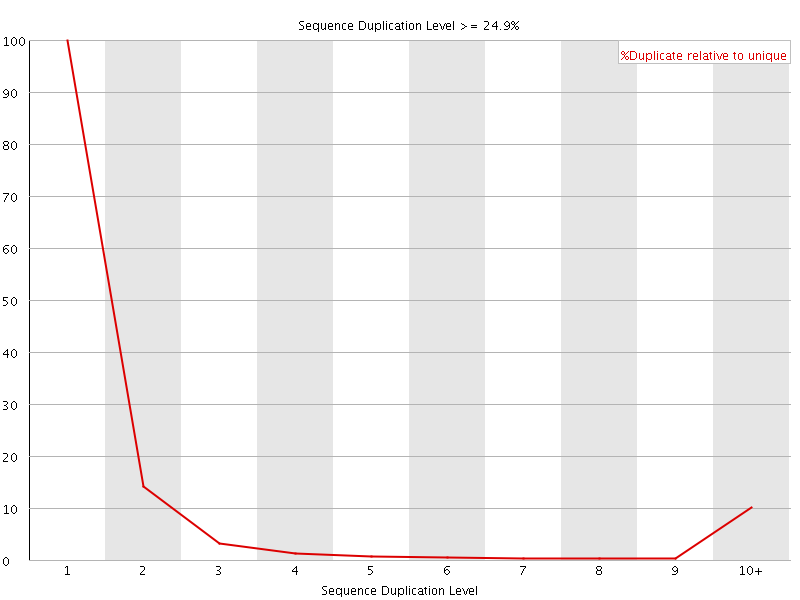

Supplement: File S1 — FastQC-generated quality plots for the datasets analyzed in this study. (ZIP) [file pone.0085024.s003.zip › fastqc/lovell_1_fastqc/Images/duplication_levels.png]

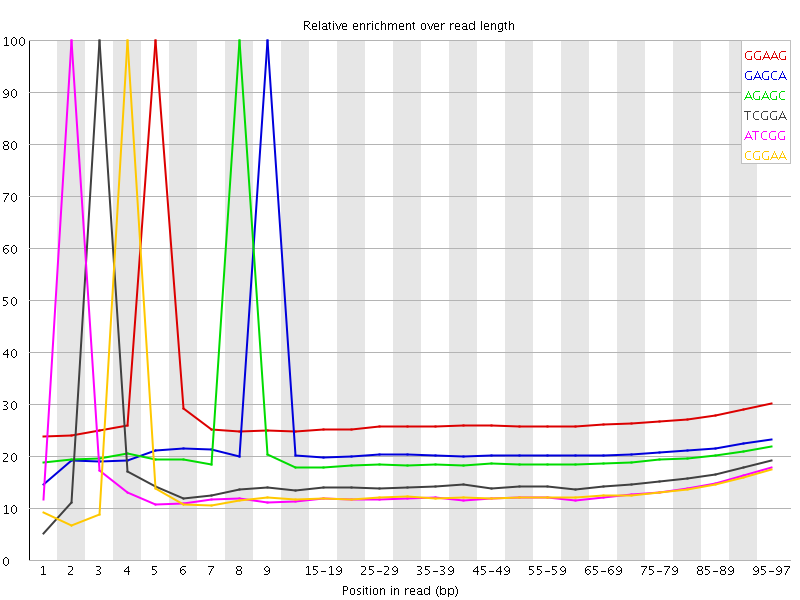

Supplement: File S1 — FastQC-generated quality plots for the datasets analyzed in this study. (ZIP) [file pone.0085024.s003.zip › fastqc/lovell_1_fastqc/Images/kmer_profiles.png]

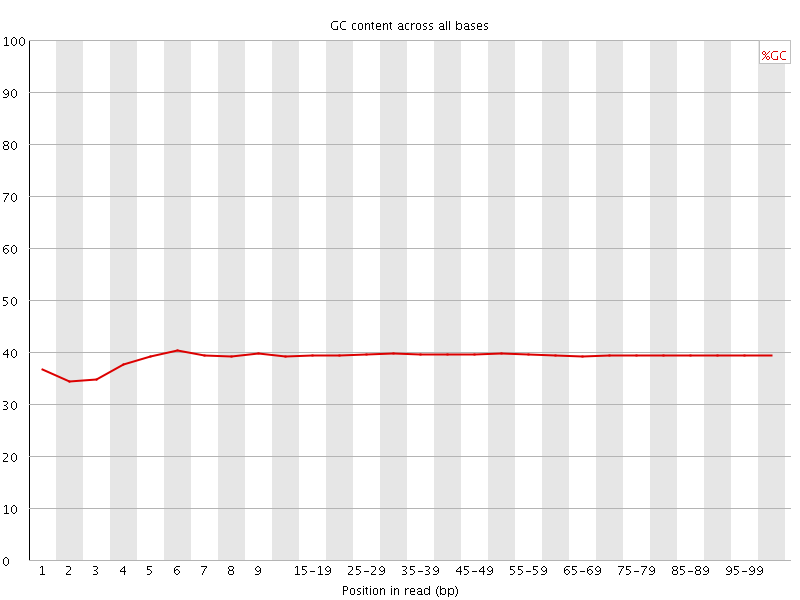

Supplement: File S1 — FastQC-generated quality plots for the datasets analyzed in this study. (ZIP) [file pone.0085024.s003.zip › fastqc/lovell_1_fastqc/Images/per_base_gc_content.png]

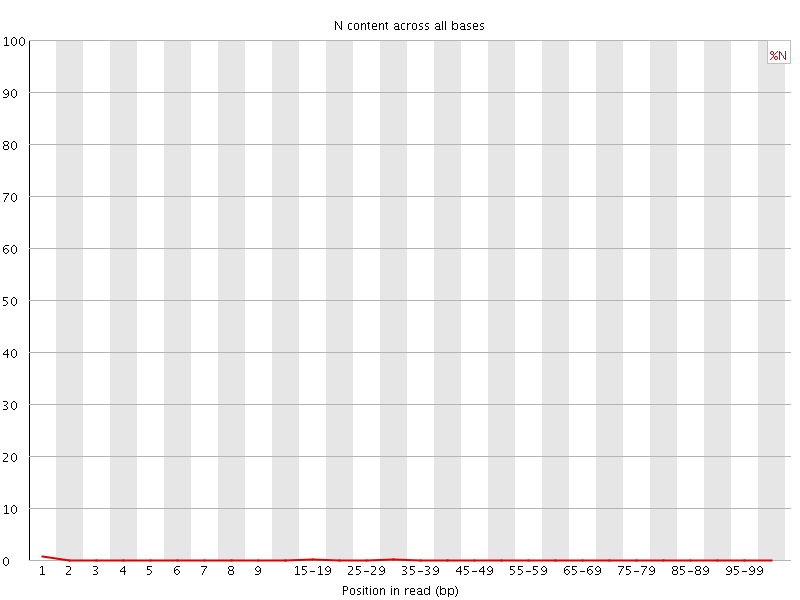

Supplement: File S1 — FastQC-generated quality plots for the datasets analyzed in this study. (ZIP) [file pone.0085024.s003.zip › fastqc/lovell_1_fastqc/Images/per_base_n_content.png]

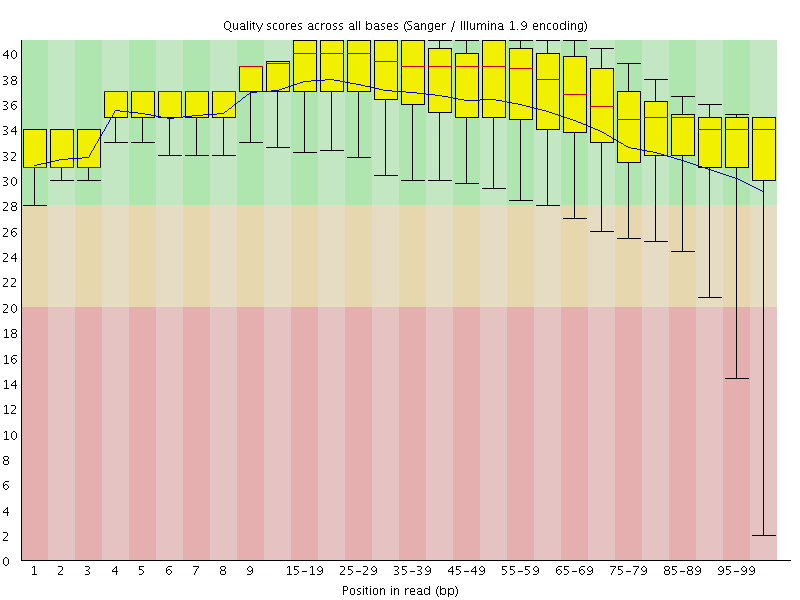

Supplement: File S1 — FastQC-generated quality plots for the datasets analyzed in this study. (ZIP) [file pone.0085024.s003.zip › fastqc/lovell_1_fastqc/Images/per_base_quality.png]

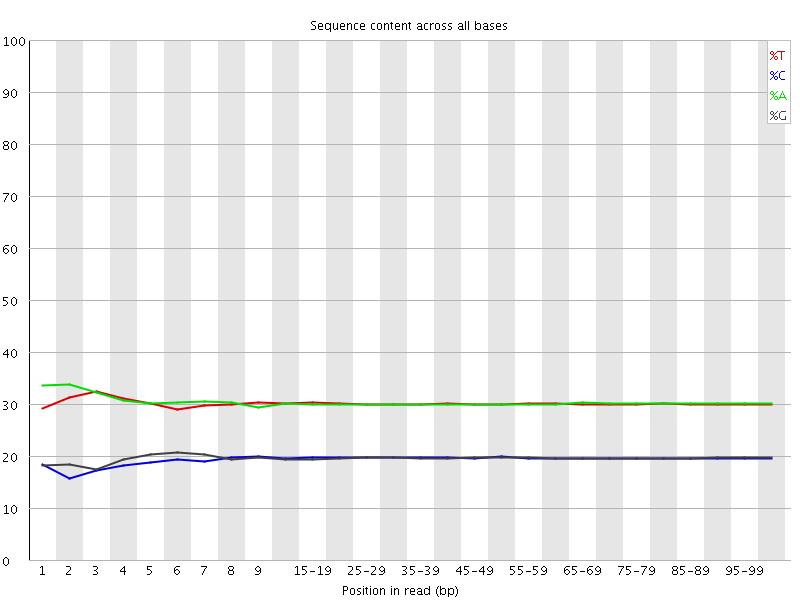

Supplement: File S1 — FastQC-generated quality plots for the datasets analyzed in this study. (ZIP) [file pone.0085024.s003.zip › fastqc/lovell_1_fastqc/Images/per_base_sequence_content.png]

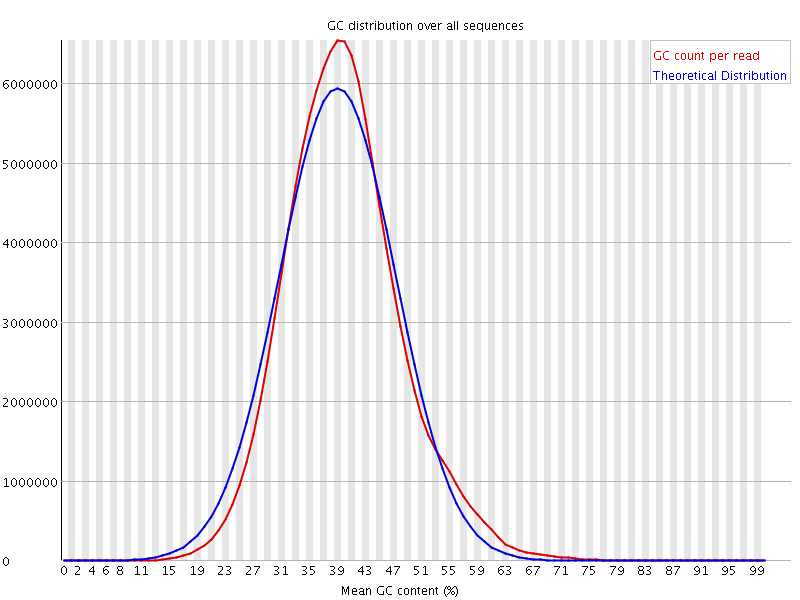

Supplement: File S1 — FastQC-generated quality plots for the datasets analyzed in this study. (ZIP) [file pone.0085024.s003.zip › fastqc/lovell_1_fastqc/Images/per_sequence_gc_content.png]

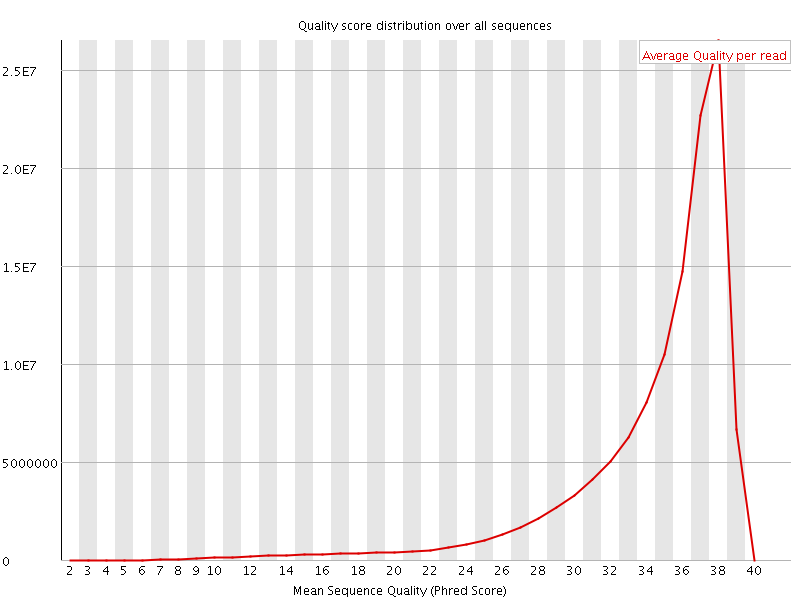

Supplement: File S1 — FastQC-generated quality plots for the datasets analyzed in this study. (ZIP) [file pone.0085024.s003.zip › fastqc/lovell_1_fastqc/Images/per_sequence_quality.png]

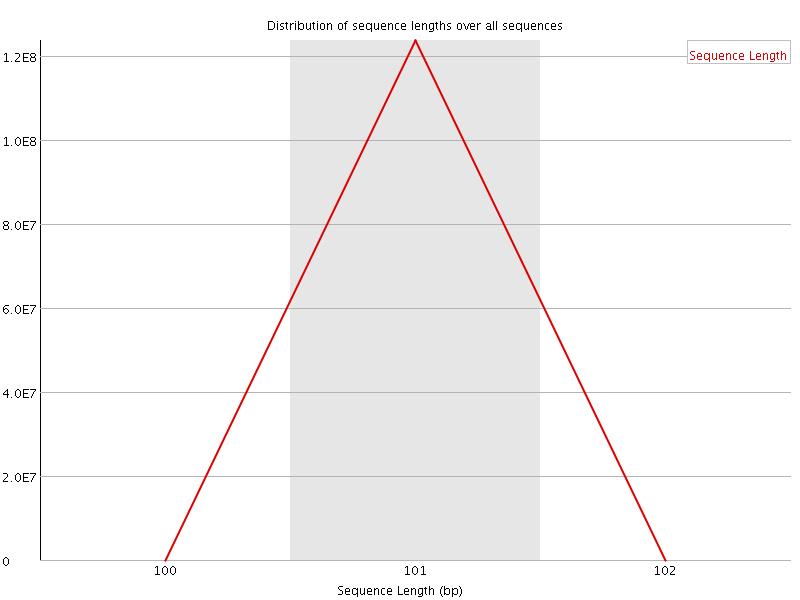

Supplement: File S1 — FastQC-generated quality plots for the datasets analyzed in this study. (ZIP) [file pone.0085024.s003.zip › fastqc/lovell_1_fastqc/Images/sequence_length_distribution.png]

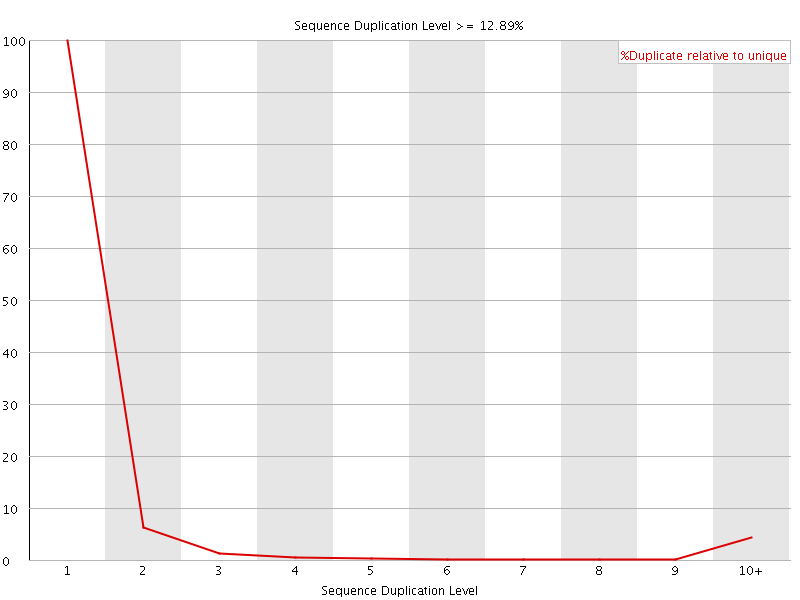

Supplement: File S1 — FastQC-generated quality plots for the datasets analyzed in this study. (ZIP) [file pone.0085024.s003.zip › fastqc/lovell_2_fastqc/Images/duplication_levels.png]

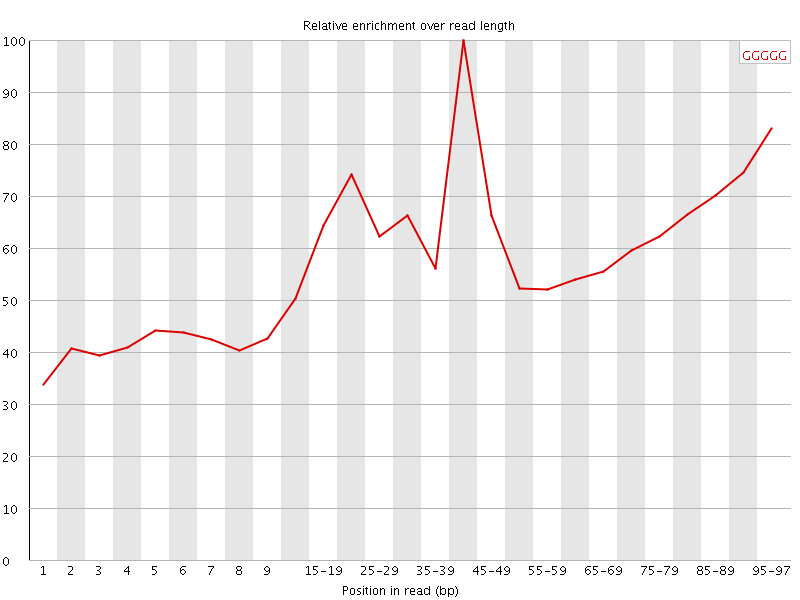

Supplement: File S1 — FastQC-generated quality plots for the datasets analyzed in this study. (ZIP) [file pone.0085024.s003.zip › fastqc/lovell_2_fastqc/Images/kmer_profiles.png]

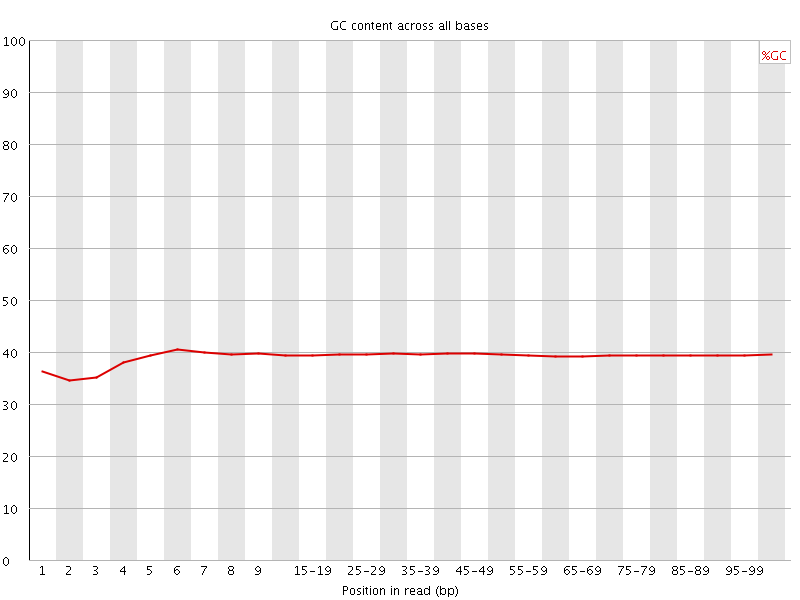

Supplement: File S1 — FastQC-generated quality plots for the datasets analyzed in this study. (ZIP) [file pone.0085024.s003.zip › fastqc/lovell_2_fastqc/Images/per_base_gc_content.png]

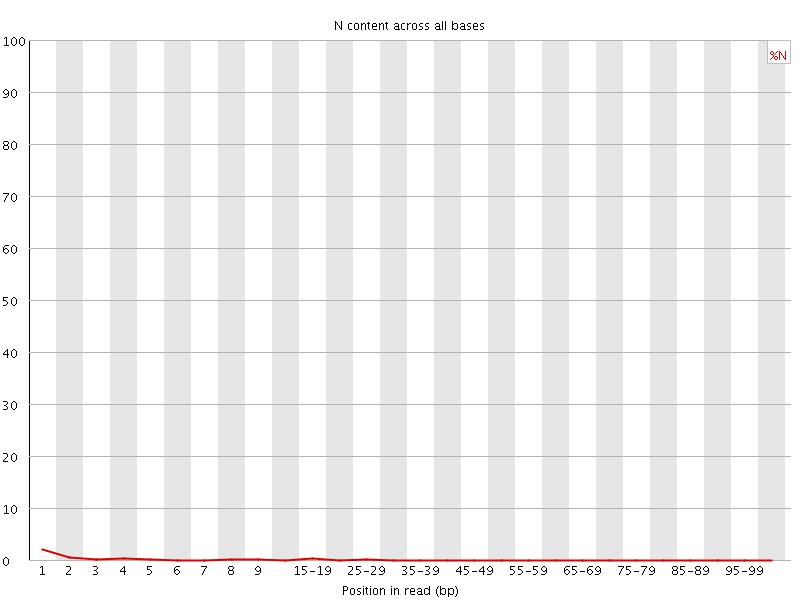

Supplement: File S1 — FastQC-generated quality plots for the datasets analyzed in this study. (ZIP) [file pone.0085024.s003.zip › fastqc/lovell_2_fastqc/Images/per_base_n_content.png]

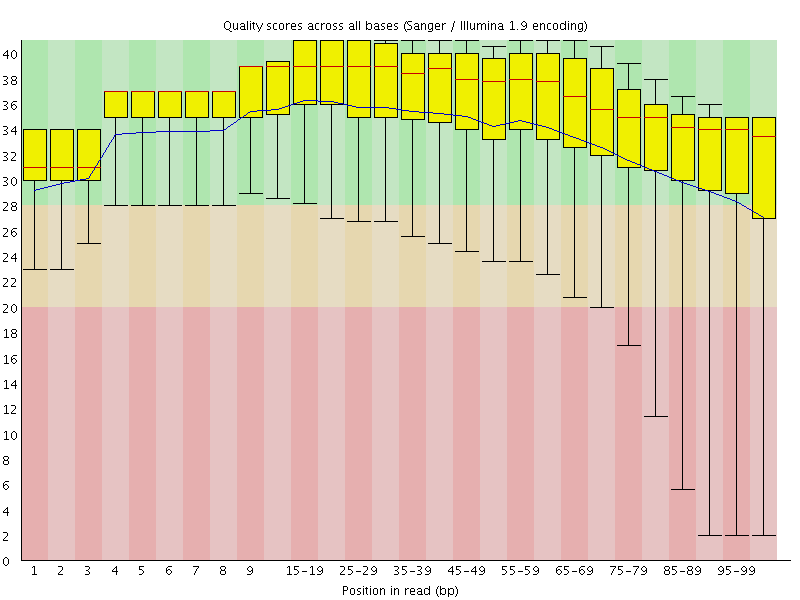

Supplement: File S1 — FastQC-generated quality plots for the datasets analyzed in this study. (ZIP) [file pone.0085024.s003.zip › fastqc/lovell_2_fastqc/Images/per_base_quality.png]

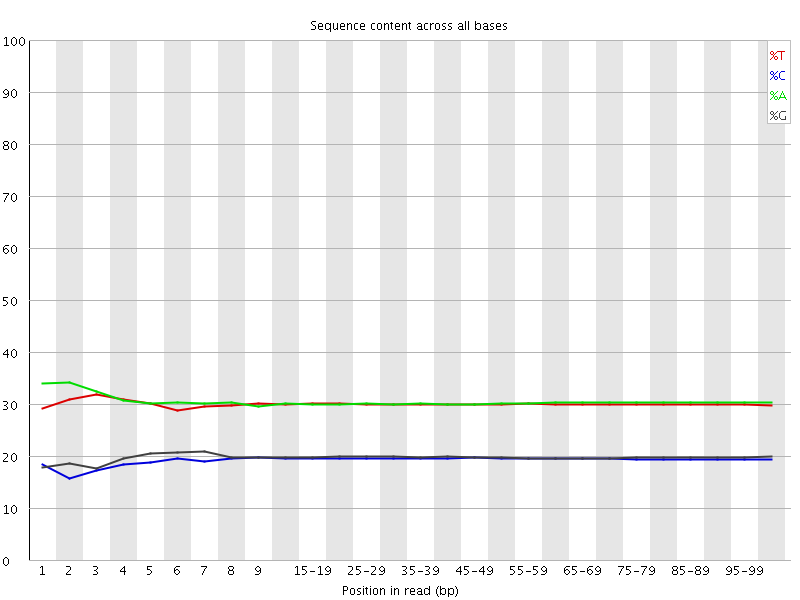

Supplement: File S1 — FastQC-generated quality plots for the datasets analyzed in this study. (ZIP) [file pone.0085024.s003.zip › fastqc/lovell_2_fastqc/Images/per_base_sequence_content.png]

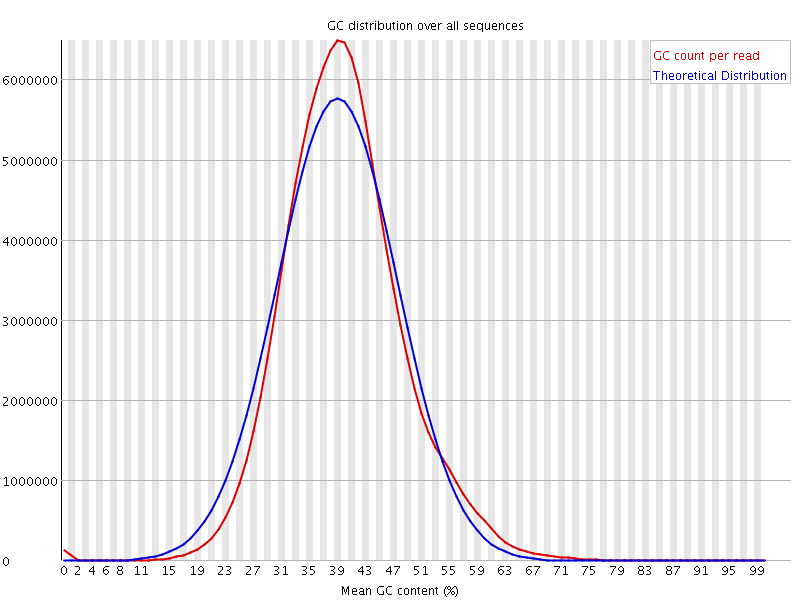

Supplement: File S1 — FastQC-generated quality plots for the datasets analyzed in this study. (ZIP) [file pone.0085024.s003.zip › fastqc/lovell_2_fastqc/Images/per_sequence_gc_content.png]

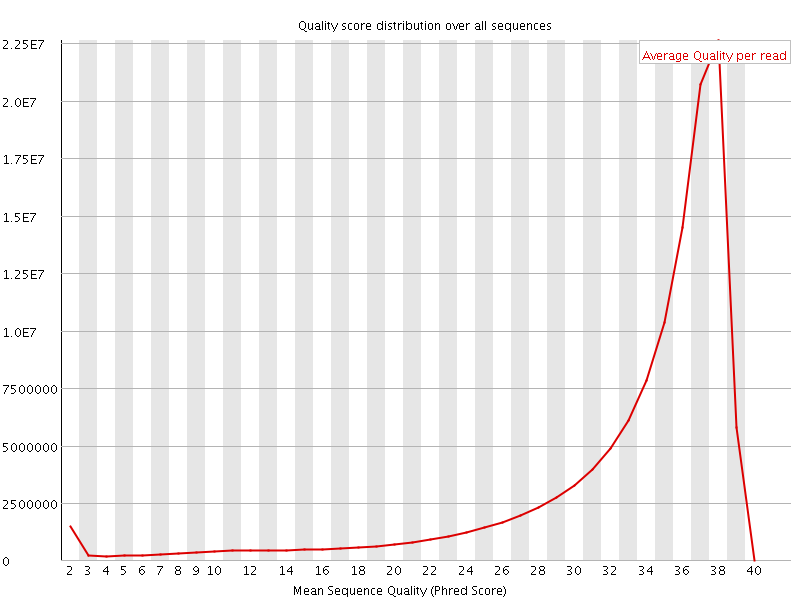

Supplement: File S1 — FastQC-generated quality plots for the datasets analyzed in this study. (ZIP) [file pone.0085024.s003.zip › fastqc/lovell_2_fastqc/Images/per_sequence_quality.png]
