# Supplementary material for: An Extensive Evaluation of Read Trimming Effects on Illumina NGS Data Analysis
Source: PLoS One. 2013 Dec 23;8(12):e85024. doi: 10.1371/journal.pone.0085024 (PMC3871669; doi:10.1371/journal.pone.0085024)
Supplement: File S1 — FastQC-generated quality plots for the datasets analyzed in this study. (ZIP) [file pone.0085024.s003.zip › fastqc/atrnaseq_SRR420813_1_fastqc/fastqc_report.html]

SRR420813\_1.fastq.gz FastQC Report


FastQC Report

Sat 1 Dec 2012  
SRR420813\_1.fastq.gz

## Summary

- Basic Statistics
- Per base sequence quality
- Per sequence quality scores
- Per base sequence content
- Per base GC content
- Per sequence GC content
- Per base N content
- Sequence Length Distribution
- Sequence Duplication Levels
- Overrepresented sequences
- Kmer Content

## Basic Statistics

| Measure | Value |
| --- | --- |
| Filename | SRR420813\_1.fastq.gz |
| File type | Conventional base calls |
| Encoding | Sanger / Illumina 1.9 |
| Total Sequences | 18583010 |
| Filtered Sequences | 0 |
| Sequence length | 83 |
| %GC | 45 |

## Per base sequence quality

## Per sequence quality scores

## Per base sequence content

## Per base GC content

## Per sequence GC content

## Per base N content

## Sequence Length Distribution

## Sequence Duplication Levels

## Overrepresented sequences

No overrepresented sequences

## Kmer Content

| Sequence | Count | Obs/Exp Overall | Obs/Exp Max | Max Obs/Exp Position |
| --- | --- | --- | --- | --- |
| TCTTC | 5159815 | 3.2896492 | 6.744436 | 7 |
| GAAGA | 4827770 | 3.2159462 | 6.9496675 | 2 |
| CTTCT | 4705190 | 2.9998024 | 6.7959223 | 1 |
| TTCTT | 5172510 | 2.7669618 | 5.3753057 | 6 |
| AAGAA | 4906880 | 2.6934378 | 6.887864 | 2 |
| GGAGA | 3097045 | 2.5036314 | 5.1880536 | 1 |
| CTTCA | 3611250 | 2.3059168 | 7.5956726 | 1 |
| CTCCT | 2943490 | 2.2366176 | 5.253295 | 1 |
| CAAGA | 3390810 | 2.2148693 | 6.5988483 | 1 |
| GGGGG | 1841050 | 2.191845 | 5.136481 | 70-74 |
| CTCCA | 2855035 | 2.1727567 | 7.5436454 | 1 |
| TCTTG | 3304005 | 2.1481953 | 5.391268 | 7 |
| GGAAG | 2562060 | 2.071153 | 5.2137485 | 1 |
| CAACA | 3227830 | 2.0674627 | 5.6031637 | 1 |
| CTTTG | 3003360 | 1.9527222 | 8.821957 | 1 |
| AAAGA | 3485330 | 1.9131341 | 5.5151086 | 2 |
| CAAAG | 2922785 | 1.9091563 | 6.392529 | 1 |
| CTTGA | 2864230 | 1.8651401 | 8.338842 | 1 |
| CTTGG | 2269895 | 1.7937835 | 9.087642 | 1 |
| AAGAT | 3149270 | 1.7260004 | 6.328969 | 3 |
| GAGAT | 2593480 | 1.7249422 | 5.401069 | 3 |
| TTGGA | 2563035 | 1.702063 | 5.5977244 | 2 |
| CTTGT | 2581825 | 1.6786487 | 5.6869593 | 1 |
| CTCTG | 2139480 | 1.657887 | 5.5513034 | 1 |
| CAAAA | 3072780 | 1.6539235 | 5.4493647 | 1 |
| TCCAA | 2583490 | 1.6522027 | 5.6804233 | 7 |
| CTCAA | 2541125 | 1.6251093 | 7.5032325 | 1 |
| TTCAA | 3010235 | 1.6152633 | 5.147775 | 7 |
| TCAAA | 2886785 | 1.5514145 | 5.099874 | 2 |
| TTTGA | 2835210 | 1.5490847 | 5.7708535 | 2 |
| AGATC | 2343700 | 1.5285378 | 6.405794 | 4 |
| GATCT | 2336315 | 1.5213704 | 5.9998555 | 5 |
| CTGGA | 1855340 | 1.468447 | 6.953933 | 1 |
| CTTTT | 2715260 | 1.4524903 | 5.2778788 | 1 |
| CTCAG | 1847520 | 1.4338588 | 8.03039 | 1 |
| TTCAC | 2243840 | 1.4327748 | 5.0582957 | 7 |
| CTCCG | 1525115 | 1.4085199 | 5.6680403 | 1 |
| GAATC | 2154065 | 1.4048597 | 5.6665845 | 4 |
| CTGAG | 1762445 | 1.3949233 | 5.629434 | 1 |
| AGATT | 2512685 | 1.3749868 | 5.291445 | 4 |
| AATCT | 2551985 | 1.3693707 | 5.246988 | 5 |
| CTGAA | 2072380 | 1.3515853 | 5.4796305 | 1 |
| ATCCA | 2062735 | 1.3191677 | 5.2397094 | 6 |
| CTCGA | 1278370 | 0.99214196 | 5.7011056 | 1 |
| CTCGT | 1247810 | 0.9669302 | 5.0202174 | 1 |
| CTCGG | 912565 | 0.8594917 | 6.0474815 | 1 |

Produced by FastQC (version 0.10.1)
