# Supplementary material for: An Extensive Evaluation of Read Trimming Effects on Illumina NGS Data Analysis
Source: PLoS One. 2013 Dec 23;8(12):e85024. doi: 10.1371/journal.pone.0085024 (PMC3871669; doi:10.1371/journal.pone.0085024)
Supplement: File S1 — FastQC-generated quality plots for the datasets analyzed in this study. (ZIP) [file pone.0085024.s003.zip › fastqc/genotyping_yeast_SRR452441_1_fastqc/fastqc_report.html]

SRR452441\_1.fastq.gz FastQC Report


FastQC Report

Fri 28 Dec 2012  
SRR452441\_1.fastq.gz

## Summary

- Basic Statistics
- Per base sequence quality
- Per sequence quality scores
- Per base sequence content
- Per base GC content
- Per sequence GC content
- Per base N content
- Sequence Length Distribution
- Sequence Duplication Levels
- Overrepresented sequences
- Kmer Content

## Basic Statistics

| Measure | Value |
| --- | --- |
| Filename | SRR452441\_1.fastq.gz |
| File type | Conventional base calls |
| Encoding | Sanger / Illumina 1.9 |
| Total Sequences | 2218610 |
| Filtered Sequences | 0 |
| Sequence length | 101 |
| %GC | 39 |

## Per base sequence quality

## Per sequence quality scores

## Per base sequence content

## Per base GC content

## Per sequence GC content

## Per base N content

## Sequence Length Distribution

## Sequence Duplication Levels

## Overrepresented sequences

| Sequence | Count | Percentage | Possible Source |
| --- | --- | --- | --- |
| GATCGGAAGAGCACACGTCTGAACTCCAGTCACGCACACGAATCTCGTAT | 10868 | 0.489856261352829 | TruSeq Adapter, Index 5 (97% over 36bp) |

## Kmer Content

| Sequence | Count | Obs/Exp Overall | Obs/Exp Max | Max Obs/Exp Position |
| --- | --- | --- | --- | --- |
| GAAGA | 675885 | 2.7543154 | 7.8571186 | 6 |
| GGAAG | 445125 | 2.7487094 | 10.531035 | 5 |
| CGGAA | 358115 | 2.2377121 | 9.976414 | 4 |
| GATCG | 341650 | 2.196822 | 9.885427 | 1 |
| ATCGG | 339500 | 2.1829975 | 10.0074835 | 2 |
| TCGGA | 321270 | 2.0657778 | 10.016575 | 3 |
| AAGAG | 506390 | 2.0636024 | 7.2443304 | 7 |
| AGAGC | 320430 | 2.0022342 | 9.989448 | 8 |
| GAGCA | 311570 | 1.9468716 | 9.944591 | 9 |
| AGATC | 430980 | 1.8287932 | 5.976074 | 95-97 |
| AGCAC | 288760 | 1.8258015 | 6.9045053 | 95-97 |
| CACAC | 274280 | 1.7548722 | 6.596697 | 95-97 |
| GCACA | 271580 | 1.7171739 | 6.7368746 | 95-97 |
| CGTCT | 209105 | 1.4000529 | 5.140115 | 95-97 |
| ACACG | 208455 | 1.3180408 | 5.8445535 | 95-97 |
| ACGTC | 201955 | 1.3140228 | 5.281158 | 95-97 |
| CACGT | 190910 | 1.2421584 | 5.446293 | 95-97 |

Produced by FastQC (version 0.10.1)
