# Supplementary material for: An Extensive Evaluation of Read Trimming Effects on Illumina NGS Data Analysis
Source: PLoS One. 2013 Dec 23;8(12):e85024. doi: 10.1371/journal.pone.0085024 (PMC3871669; doi:10.1371/journal.pone.0085024)
Supplement: File S1 — FastQC-generated quality plots for the datasets analyzed in this study. (ZIP) [file pone.0085024.s003.zip › fastqc/hsrnaseq_SRR002073_1_fastqc/fastqc_report.html]

SRR002073\_1.fastq.gz FastQC Report


FastQC Report

Fri 28 Dec 2012  
SRR002073\_1.fastq.gz

## Summary

- Basic Statistics
- Per base sequence quality
- Per sequence quality scores
- Per base sequence content
- Per base GC content
- Per sequence GC content
- Per base N content
- Sequence Length Distribution
- Sequence Duplication Levels
- Overrepresented sequences
- Kmer Content

## Basic Statistics

| Measure | Value |
| --- | --- |
| Filename | SRR002073\_1.fastq.gz |
| File type | Conventional base calls |
| Encoding | Sanger / Illumina 1.9 |
| Total Sequences | 9104944 |
| Filtered Sequences | 0 |
| Sequence length | 33 |
| %GC | 50 |

## Per base sequence quality

## Per sequence quality scores

## Per base sequence content

## Per base GC content

## Per sequence GC content

## Per base N content

## Sequence Length Distribution

## Sequence Duplication Levels

## Overrepresented sequences

| Sequence | Count | Percentage | Possible Source |
| --- | --- | --- | --- |
| AAAAAAAAAAAAAAAAAAAAAAAAAAAAAAAAA | 56163 | 0.6168406966588702 | No Hit |
| GNNNNNNNNNNNNNNNNNNNNNNNNNNNNNNNN | 31030 | 0.3408038533789993 | No Hit |
| CNNNNNNNNNNNNNNNNNNNNNNNNNNNNNNNN | 29630 | 0.3254275918665727 | No Hit |
| TNNNNNNNNNNNNNNNNNNNNNNNNNNNNNNNN | 17427 | 0.19140150669789951 | No Hit |
| ANNNNNNNNNNNNNNNNNNNNNNNNNNNNNNNN | 15380 | 0.16891921575794425 | No Hit |

## Kmer Content

| Sequence | Count | Obs/Exp Overall | Obs/Exp Max | Max Obs/Exp Position |
| --- | --- | --- | --- | --- |
| AAAAA | 2685280 | 10.89772 | 16.35504 | 2 |
| CCAGG | 516030 | 2.0868125 | 5.2367606 | 8 |
| CTTCA | 501320 | 2.0425448 | 5.4286118 | 6 |
| CCAGC | 491430 | 1.9748309 | 5.197434 | 8 |
| AAAAT | 469575 | 1.9171709 | 6.541483 | 3 |
| CTCCA | 474505 | 1.9139606 | 7.44265 | 6 |
| GAAAA | 457855 | 1.8623432 | 6.276263 | 1 |
| TCCAG | 458295 | 1.8602773 | 7.612678 | 7 |
| CCACC | 465790 | 1.8600221 | 5.90751 | 8 |
| TCCTG | 453140 | 1.850439 | 5.1429067 | 7 |
| TTTCA | 418305 | 1.7215277 | 5.021953 | 1 |
| TTCCA | 403685 | 1.6447474 | 5.216775 | 6 |
| TCTGC | 378570 | 1.5459255 | 5.186833 | 7 |
| TCCAC | 358030 | 1.444148 | 6.1705093 | 7 |
| TCCAA | 310530 | 1.2576226 | 5.4231124 | 7 |
| ATCCA | 290555 | 1.1767254 | 7.0173388 | 6 |
| AAATC | 276800 | 1.1255567 | 5.1984224 | 4 |

Produced by FastQC (version 0.10.1)
