# Supplementary material for: An Extensive Evaluation of Read Trimming Effects on Illumina NGS Data Analysis
Source: PLoS One. 2013 Dec 23;8(12):e85024. doi: 10.1371/journal.pone.0085024 (PMC3871669; doi:10.1371/journal.pone.0085024)
Supplement: File S1 — FastQC-generated quality plots for the datasets analyzed in this study. (ZIP) [file pone.0085024.s003.zip › fastqc/lovell_1_fastqc/fastqc_report.html]

lovell\_1.fastq.gz FastQC Report


FastQC Report

Sat 26 Jan 2013  
lovell\_1.fastq.gz

## Summary

- Basic Statistics
- Per base sequence quality
- Per sequence quality scores
- Per base sequence content
- Per base GC content
- Per sequence GC content
- Per base N content
- Sequence Length Distribution
- Sequence Duplication Levels
- Overrepresented sequences
- Kmer Content

## Basic Statistics

| Measure | Value |
| --- | --- |
| Filename | lovell\_1.fastq.gz |
| File type | Conventional base calls |
| Encoding | Sanger / Illumina 1.9 |
| Total Sequences | 123590441 |
| Filtered Sequences | 0 |
| Sequence length | 101 |
| %GC | 39 |

## Per base sequence quality

## Per sequence quality scores

## Per base sequence content

## Per base GC content

## Per sequence GC content

## Per base N content

## Sequence Length Distribution

## Sequence Duplication Levels

## Overrepresented sequences

No overrepresented sequences

## Kmer Content

| Sequence | Count | Obs/Exp Overall | Obs/Exp Max | Max Obs/Exp Position |
| --- | --- | --- | --- | --- |
| GGAAG | 15337880 | 1.8091838 | 6.674472 | 5 |
| GAGCA | 11128750 | 1.3163522 | 6.1449485 | 9 |
| AGAGC | 10143580 | 1.1998224 | 6.0397167 | 8 |
| TCGGA | 7345905 | 0.87132096 | 5.6004643 | 3 |
| ATCGG | 6405195 | 0.7597404 | 5.556747 | 2 |
| CGGAA | 6181170 | 0.73113304 | 5.381553 | 4 |
| GATCG | 5725135 | 0.6790763 | 5.567729 | 1 |

Produced by FastQC (version 0.10.1)
