# Supplementary material for: An Extensive Evaluation of Read Trimming Effects on Illumina NGS Data Analysis
Source: PLoS One. 2013 Dec 23;8(12):e85024. doi: 10.1371/journal.pone.0085024 (PMC3871669; doi:10.1371/journal.pone.0085024)
Supplement: File S1 — FastQC-generated quality plots for the datasets analyzed in this study. (ZIP) [file pone.0085024.s003.zip › fastqc/lovell_2_fastqc/fastqc_report.html]

lovell\_2.fastq.gz FastQC Report


FastQC Report

Sat 26 Jan 2013  
lovell\_2.fastq.gz

## Summary

- Basic Statistics
- Per base sequence quality
- Per sequence quality scores
- Per base sequence content
- Per base GC content
- Per sequence GC content
- Per base N content
- Sequence Length Distribution
- Sequence Duplication Levels
- Overrepresented sequences
- Kmer Content

## Basic Statistics

| Measure | Value |
| --- | --- |
| Filename | lovell\_2.fastq.gz |
| File type | Conventional base calls |
| Encoding | Sanger / Illumina 1.9 |
| Total Sequences | 123590441 |
| Filtered Sequences | 0 |
| Sequence length | 101 |
| %GC | 39 |

## Per base sequence quality

## Per sequence quality scores

## Per base sequence content

## Per base GC content

## Per sequence GC content

## Per base N content

## Sequence Length Distribution

## Sequence Duplication Levels

## Overrepresented sequences

No overrepresented sequences

## Kmer Content

| Sequence | Count | Obs/Exp Overall | Obs/Exp Max | Max Obs/Exp Position |
| --- | --- | --- | --- | --- |
| GGGGG | 11500510 | 3.097964 | 4.954914 | 40-44 |

Produced by FastQC (version 0.10.1)
