# Supplementary material for: An Extensive Evaluation of Read Trimming Effects on Illumina NGS Data Analysis
Source: PLoS One. 2013 Dec 23;8(12):e85024. doi: 10.1371/journal.pone.0085024 (PMC3871669; doi:10.1371/journal.pone.0085024)
Supplement: File S2 — Comparative analysis of the overlaps of the trimming tools investigated in the current paper. (DOCX) [file pone.0085024.s004.docx]

## Supplementary File S2

## “An extensive evaluation of read trimming effects on Illumina NGS data analysis”

Del Fabbro, C.^1,☨^ ; Scalabrin, S.^2,☨^, Morgante, M.^1^ and Giorgi F.M.^1,3*^

^1^ Institute of Applied Genomics, 33100, Udine, Italy

^2^ IGA Technology Services, 33100, Udine, Italy

^3^ Center for Computational Biology and Bioinformatics, Columbia University, 10032, New York, U.S.A.

^*^To whom correspondence should be addressed

^☨^ These authors contributed equally

### General analysis of trimmer overlaps

Similarities and differences not immediately evident by the pure examination of the trimmer’s algorithms were assessed by an unsupervised comparison of the different tools in all the four datasets. We performed a pairwise comparison counting the commonly trimmed nucleotide by each pair of tools.

The comparison was made processing outputs obtained with quality value Q set to 20 for each tool (ConDeTri requires two values, hence we have used its default values: HQ=25 and LQ=10). In Supplementary Tables 1-4 we reported the percentage of common bases, normalized by the total trimmed bases by each tool (last column) for every dataset. We have obtained a non-symmetric matrix where each cell represents the percentage of bases trimmed by one tool in common with all bases trimmed by a second tool. By a visual inspection of the matrix we have inferred the relationships summarized in Supplementary Figure 1. In the figure we show relationship among the trimmers for each dataset, in a graph-based representation of inclusion. For instance, the arrow from ConDeTri to Sickle in Supplementary Figure 1(C) means that at least 90% of the bases trimmed by Sickle are trimmed also by ConDeTri, but the vice versa does not hold. Instead, the circle surrounding Prinseq and FASTX in Supplementary Figure 1(B) means that 90% of the bases trimmed by Prinseq are in common with FASTX and the vice versa also hold (not surprising because the algorithm is the same). The same behavior is evident for SolexaQA-BWA and Cutadapt that implement the same algorithm.

More interesting, most evident in sub-figures (A) and (C) but notable also in (B), five trimmers are roughly equivalent and they trim out, in most cases, the same bases. Surprising these trimmers implement algorithm “crossing” our naïve algorithm subdivision: three are running sum based and two are window based algorithms. In the sub-figure (D) this correlation are less evident and, due to the high divergence of quality scores for Human reads, the peculiarities of each tool became significant.


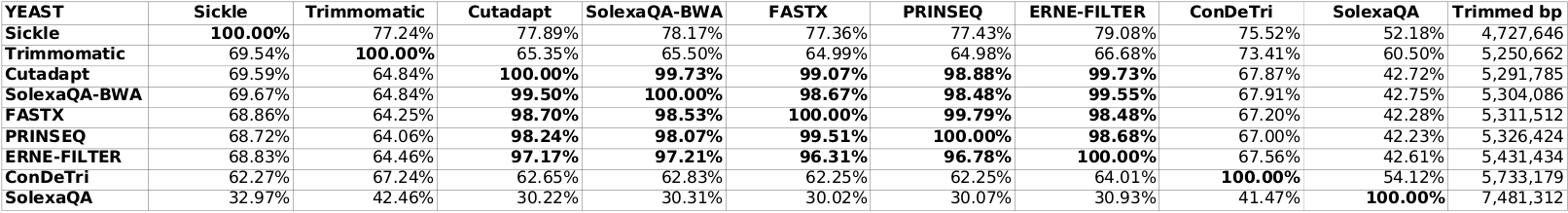


Table 1. Yeast’s overlap values. Percentage greater than 90% are bolded.


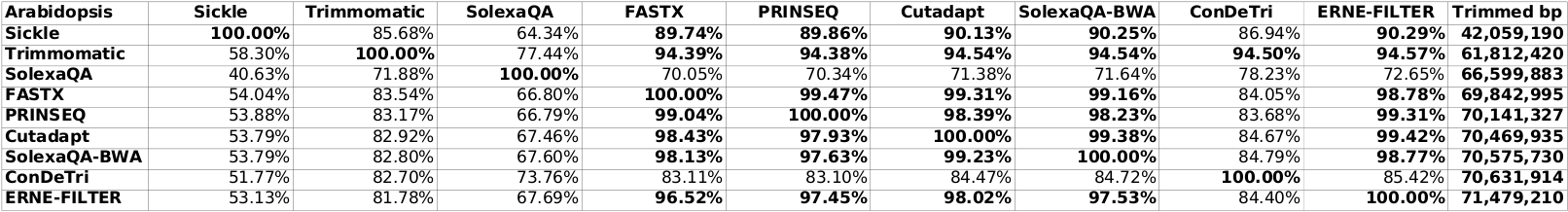


Table 2. Arabidopsis’s overlap values. Percentage greater than 90% are bolded.


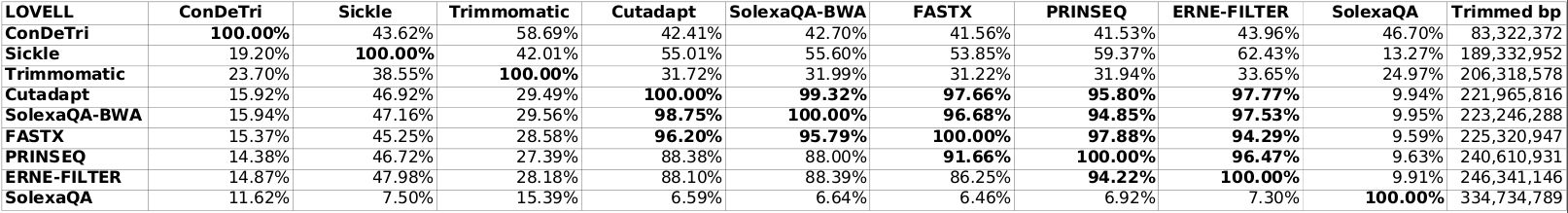


Table 3. Lovell’s overlap values. Percentage greater than 90% are bolded.


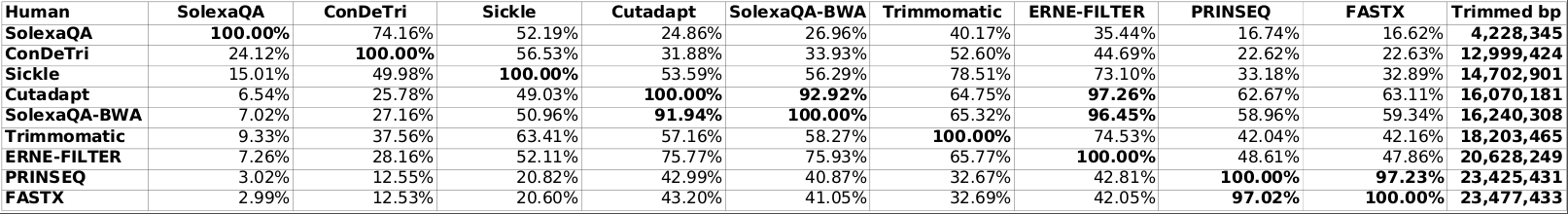


Table 4. Human’s overlap values. Percentage greater than 90% are bolded.


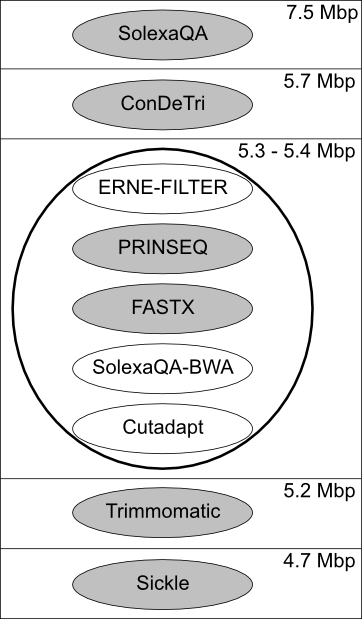

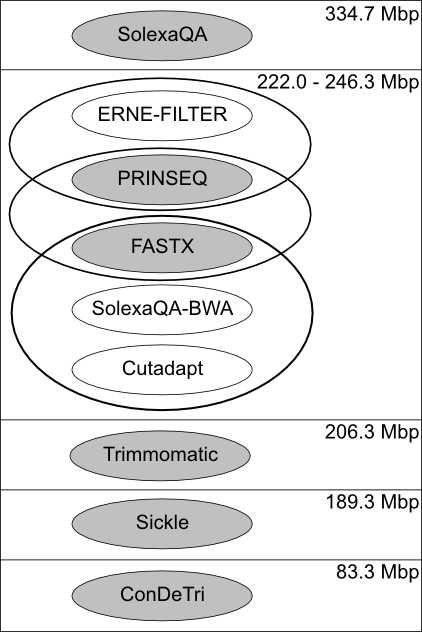


(A) Yeast (B) Lovell


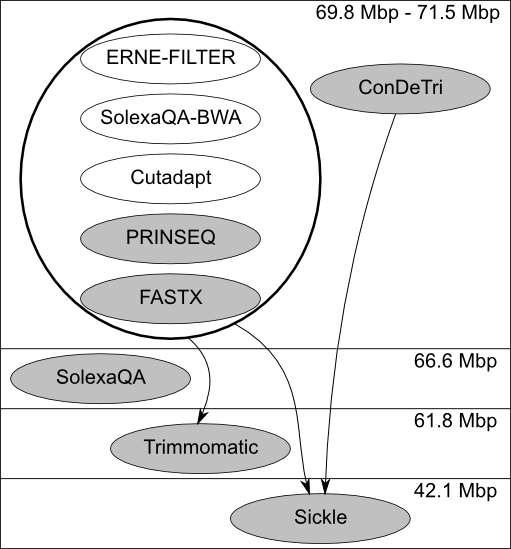

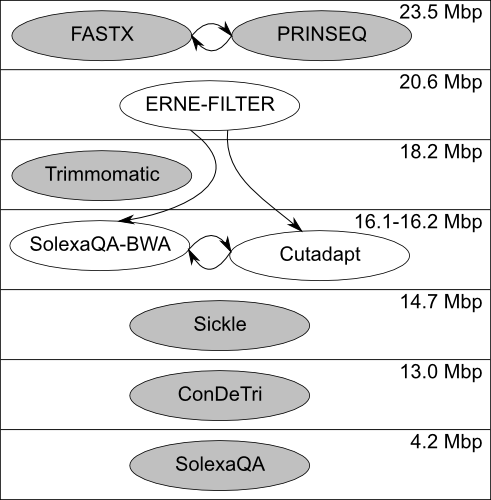


(C) Arabidopsis (D) Human

Figure 1. Trimmers relationship: an arrow from X to Y (X→ Y) means that Y is “included” in X. Y is included in X if and only if at least 90% of trimmed bases by B is also trimmed by A. Each box represents an amount of trimmed bases, indicate in the top-right corner of each box. Gray backgrounds represent window based tools, while white backgrounds represent running sums based programs. If a pair (or more) of trimmers can be connected with a bidirectional arrow (hence X→ Y and Y→ X) they are collapsed together in a surrounding circle.
